# TALEN mediated gene editing in a mouse model of Fanconi anemia

**DOI:** 10.1038/s41598-020-63971-z

**Published:** 2020-04-24

**Authors:** Maria José Pino-Barrio, Yari Giménez, Mariela Villanueva, Marcus Hildenbeutel, Rebeca Sánchez-Dominguez, Sandra Rodríguez-Perales, Roser Pujol, Jordi Surrallés, Paula Río, Toni Cathomen, Claudio Mussolino, Juan Antonio Bueren, Susana Navarro

**Affiliations:** 1 Division of Hematopoietic Innovative Therapies, CIEMAT/CIBERER, 28040 Madrid, Spain; 2 Advanced Therapies Unit, IIS-Fundación Jimenez Diaz (IIS-FJD, UAM), 28040 Madrid, Spain; 3 Center for Biomedical Network Research on Rare Diseases, Instituto de Salud Carlos III, Madrid, Spain; 4 Molecular Cytogenetics Group, Human Cancer Genetics Program, Centro Nacional de Investigaciones Oncológicas (CNIO), Melchor Fernandez Almagro, 3, 28029 Madrid, Spain; 5 Genome Instability and DNA Repair Group, Department of Genetics and Microbiology, Universitat Autònoma de Barcelona, 08193 Barcelona, Spain; 6 Institute for Transfusion Medicine and Gene Therapy, Medical Center - University of Freiburg, 79106 Freiburg, Germany; 7 Center for Chronic Immunodeficiency, Medical Center - University of Freiburg, 79106 Freiburg, Germany; 8 Faculty of Medicine, University of Freiburg, Freiburg, Germany

**Keywords:** Targeted gene repair, DNA recombination

## Abstract

The promising ability to genetically modify hematopoietic stem and progenitor cells by precise gene editing remains challenging due to their sensitivity to *in vitro* manipulations and poor efficiencies of homologous recombination. This study represents the first evidence of implementing a gene editing strategy in a murine *safe harbor* locus site that phenotypically corrects primary cells from a mouse model of Fanconi anemia A. By means of the co-delivery of transcription activator-like effector nucleases and a donor therapeutic FANCA template to the *Mbs85* locus, we achieved efficient gene targeting (23%) in mFA-A fibroblasts. This resulted in the phenotypic correction of these cells, as revealed by the reduced sensitivity of these cells to mitomycin C. Moreover, robust evidence of targeted integration was observed in murine wild type and FA-A hematopoietic progenitor cells, reaching mean targeted integration values of 21% and 16% respectively, that were associated with the phenotypic correction of these cells. Overall, our results demonstrate the feasibility of implementing a therapeutic targeted integration strategy into the m*Mbs85* locus, ortholog to the well-validated *hAAVS1*, constituting the first study of gene editing in mHSC with TALEN, that sets the basis for the use of a new *safe harbor* locus in mice.

## Introduction

Fanconi anemia (FA) is a rare genetic disorder associated with mutations in any of the twenty-two FA genes, known as FANC genes^1,2^. The genetic products of these genes belong to a DNA repair pathway known as the FA/BRCA pathway, which is involved in the repair of interstrand cross-link (ICL) lesions. FA patient cells are characterized by the accumulation of DNA damage at an increased rate as compared to healthy cells, due to an ineffective FA/BRCA DNA repair pathway. Furthermore, most patients show congenital abnormalities at birth, cancer predisposition^3–5^, and bone marrow failure^6,7^. Due to the risks associated to allogeneic hematopoietic stem cell transplantation, alternative curative treatments have been proposed. This is the case of gene therapy approaches which aim at the correction of autologous hematopoietic stem and progenitor cells (HSPC) with therapeutic lentiviral vectors. The efficiency and safety of these strategies in preclinical stages have previously been demonstrated^8–13^ and are nowadays tested in clinical trials^14–19^. Targeted gene therapy approaches are evolving as a promising new alternative approaches to avoid random integration issues arising from the use of integrative vectors.

Due to the fact that the most frequent complementation group in FA patients is FA-A (60–70%), and given that mutations in FANCA are highly heterogeneous^20–22^, a therapeutic strategy to precisely insert a therapeutic *FANCA* expression cassette into a *safe harbor* locus^23,24^ would be applicable to all *FANCA* mutations^25,26^ and could be in principle extended to other FA subtypes.

The human *AAVS1* locus located on the first intron of the *MBS85 (*PPP1R12C) gene on chromosome 19^27^ meets the requirements of a *safe harbor* locus in a wide range of cell types^28–34^. It has an open chromatin status and also contains a putative insulator element^35^. Thus, based on the favorable results observed in human cells *in vitro*, we explored a similar strategy in the context of a mouse model of FA-A by integrating a therapeutic FANCA expression cassette into the murine *Mbs85* gene, the ortholog of the human *AAVS1*^36–39^. This mouse *Mbs85* locus is located in the reverse strand of chromosome 7. The gene spans 20 kilobases (kb), and the resulting 3.1 kb mouse cDNA and protein are 77% and 86% identical to their human counterparts, respectively^40^. Importantly, the gene is located >2.5 kilobases (kb) from the 5′ end of any coding region, and the target site has been designed outside any coding region, in an intron site whose ablation is well tolerated^41^. These observations, together with the stable expression of fluorescent and therapeutic transgene inserted in this locus, and the phenotype correction demonstrated in human FA cells, would set the basics of the requirements of a *safe harbor* locus to be used for the gene therapy (GT) of FA.

Our study demonstrates the feasibility of conducting a targeted gene therapy approach in embryonic fibroblasts and hematopoietic progenitors from a mouse model of FA-A using TALE nucleases as a gene editing platform, and highlights the potential of using for the first time the *Mbs85* locus as a murine *safe harbor* for targeted integration, opening a new platform for *in vitro* and *in vivo* gene therapy applications in different disease models.

## Results

### Targeted genome integration in FA-A mouse embryonic fibroblasts

**Figure 1:**
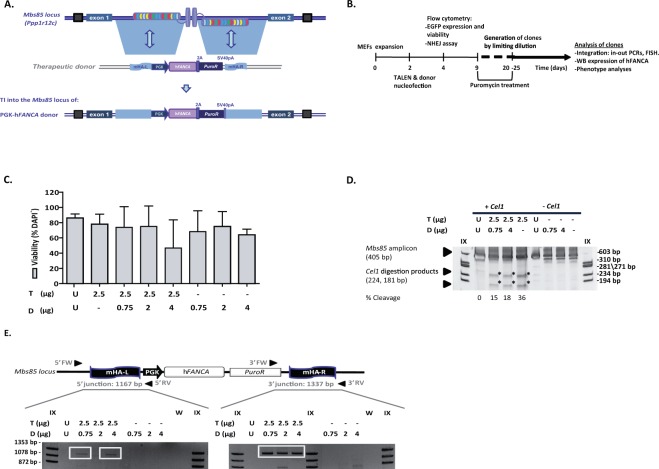
*Mbs85*-specific TALEN and donor-mediated targeted insertion in FA MEFs. (**a**) Schematic showing the architecture of the murine *Mbs85* locus, with the target sites of the TALENs highlighted, and the structure of the donor used with the therapeutic h*FANCA* cassette driven by the phosphoglycerate kinase promoter (PGK) flanked by sequences homologous to the genomic target locus. The resulting locus upon targeted integration (TI) of the donor is indicated in the lowest part of the panel. mHA-L and mHA-R: homology arms to the murine *Mbs85* locus; 2A: 2A self-cleaving peptide sequence; SV40pA: simian virus 40 polyA sequence; *PuroR*: Puromycin resistance gene. (**b**) Flow chart indicating the study design and the different analyses performed in gene-edited cells of FA-A MEFs. (**c**) Analysis of viability (percentage of DAPI^−^ cells) in the different conditions. U: untransfected cells; T: 2.5 µg of each TALEN monomer; D: donor doses (0.75, 2 or 4 µg). Bars indicate the mean ± S.D. (n = 2 experiments). d) Cleavage efficacy of the *Mbs85*-specific TALENs analysed by Surveyor assay. Representative electrophoresis gel showing the disruption of the target locus in FA-A MEFs nucleofected with only the TALENs (T, 2.5 µg of each TALEN monomer) or together with different donor doses (0.75 µg and 4 µg). U: untransfected cells. Samples not digested with the *Cel1* endonuclease were used as controls. The extent of TALEN cleavage, measured as the mean percentage of modified alleles, is indicated below. Arrows indicate the size of the parental band (405 bp) and the expected positions of the digestion products (224 bp and 181 bp), that are also indicated with asterisks. IX: DNA molecular weight marker. e) Schematic representation of the targeted integration of the therapeutic PGK-*hFANCA* donor into the *Mbs85* locus of FA-A MEFs. Arrows represent the primers, forward (Fw) and reverse (Rv) used to evaluate the site-specific integration and the size of the PCR amplicon is indicated for each integration junctions. The electrophoresis gel below is a representative image of the integration analysis performed on the same samples as indicated in (**c**). W: water control; IX: DNA molecular weight marker.

To establish efficient genome editing at the murine ortholog of the human *AAVS1* gene, we generated a pair of transcription activator-like effector nucleases (TALEN)^42^ targeting the first intron of the murine *Mbs85* gene (Supplementary Fig. 1a). The expression of each TALEN monomer was then evaluated by western blot analyses in HEK-293T cells upon lipofection of the corresponding plasmids using an antibody recognizing the HA-tag (Supplementary Fig. 1b). Mouse embryonic fibroblasts (MEFs) from *Fanca*^−/−^ mice were used to define the optimal conditions for achieving targeted integration at the murine *Mbs85* locus. *Mbs85*-specific TALENs and a donor construct containing a therapeutic human *FANCA* cassette flanked by homologous sequences to the murine *Mbs85* gene (Fig. 1a,b) were delivered into the FA-A MEFs via nucleofection using different amounts of the *FANCA* therapeutic donor and a fixed dose of TALEN monomers expression plasmids (Fig. 1c**)**.

The viability of nucleofected cells was on average around 69% in comparison with 86% of untransfected cells at 48 hours post nucleofection (Fig. 1c). An *EGFP* control plasmid was nucleofected in these cells as a control to evaluate the transfection efficiency in MEFs, that resulted in 30.17 ± 6.15%. Then, we analyzed the ability of the designed TALEN to disrupt the mouse *Mbs85* locus using Surveyor assay. The frequency of indel mutations ranged from 15% to 36% after nucleofection with these TALENs (Fig. 1d). The simultaneous delivery of TALENs and donor DNA reduced the frequency of indel mutations by 50%, as compared to delivering the nucleases alone (Fig. 1d, conditions T + D), suggesting that a number of DSBs were repaired by HR instead of NHEJ.

Once the ability of designed TALENs to cleave the *Mbs85* locus had been corroborated, we evaluated the efficacy of the gene targeting strategy. The presence of a puromycin selection marker in the therapeutic donor allowed us to enrich the population of FA-A MEFs that underwent correct gene targeting. Nucleofected cells were maintained in culture for 5 passages in the presence of puromycin (1–1.25 µg/ml). Diagnostic PCRs were then performed in the bulk population to demonstrate the integration of the therapeutic PGK-h*FANCA* donor into the *Mbs85* locus using the primers indicated to amplify the 5′ and the 3′ integration junctions (Fig. 1e and Supplementary Table 4). Samples nucleofected with the TALENs and donor revealed successful gene targeting, with amplification of the specific bands corresponding to the insertion of the donor into the *Mbs85* locus (as shown in Fig. 1e).

A total of 174 clones were generated from one single cell by limiting dilution with the aim of determining the efficiency of targeted integration in FA-A MEFs. In two independent experiments, a mean of 23.1% of the clones showed site-specific gene targeting in the *Mbs85* locus, reaching a maximum of 7.4% efficiency at the 5′ integration junction. Overall, five clones (nucleofected with 2.5 µg of each TALEN monomer and 0.75 µg of the therapeutic donor) showed the expected 5′ and 3′ integration junctions.

**Figure 2:**
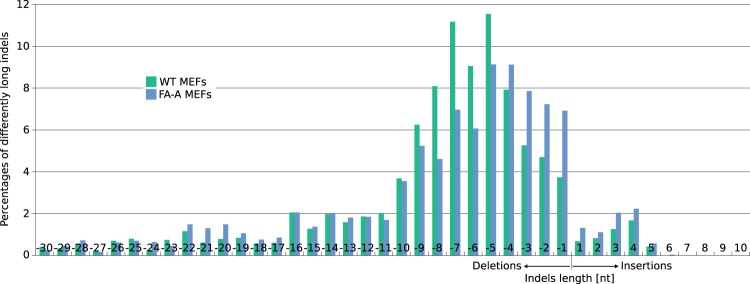
Differential length of nuclease-induced indels in WT vs. FA-A MEFs. All single deletions and insertions at the on-target site were analyzed for their length in nuclease treated WT- and FA-A MEF cells. The percentage of the different deletions or insertions was calculated in respect to all deletions or insertions respectively. The range shown was reduced to deletions with a maximal length of −30 nt and insertions with a maximal length of 10 nt.

Subsequently, we proceeded to determine the frequencies of insertion/deletion (indel) mutations in the FA-MEFs pool by deep sequencing at the on-target site and the 23 putative off-target sites predicted by PROGNOS^43^ (Supplementary Tables 1 and 2). The frequency of TALEN-induced indels at the on-target locus of WT and FA-A MEFs was 37.1%, and 30.1%, respectively. These frequencies were also confirmed by Surveyor assay (Supplementary Fig. 1c). Importantly, the frequencies of indels at three identified off-target sites ranged from 0.06% to 0.36%, showing that the used TALENs were highly specific (Supplementary Tables 1 and 3). Interestingly, while in WT MEFs we observed deletions with medium lengths of 5 to 9 nucleotides, shorter deletions of 1 to 4 nucleotides were found in FA-A MEFs (Fig. 2). A more detailed analysis of the different indels generated at the on-target site (Supplementary Figs. 2 and 3) revealed subtle differences between samples of WT or FA-A MEFs, suggesting a potential different repair preference upon nuclease treatment in the two cell types. Furthermore, these analyses revealed that the on-target cleavage predominantly occurred in the range of nucleotides 222 to 226 (Supplementary Fig. 4).

### Generation of disease-free and functional FA-A MEF clones corrected by gene targeting in the *Mbs85* locus

**Figure 3:**
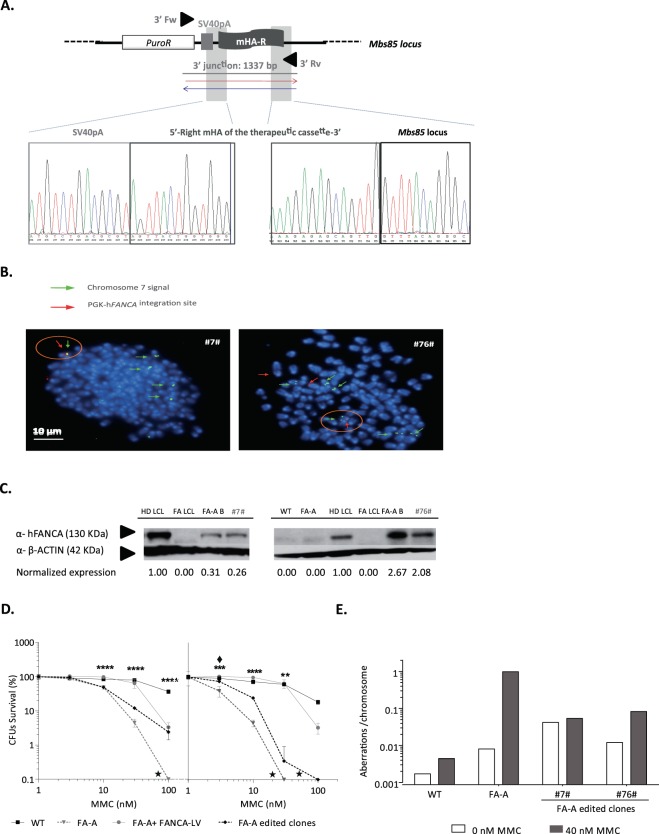
Targeted integration of the therapeutic h*FANCA* donor into the *Mbs85* locus and phenotypic correction of gene-edited FA-A MEF clones #7 and #76#. (**a**) Sequenced region of clone #76# displaying the chromatogram containing the SV40pA sequence, the left and right mHA, and the right location of the *Mbs85* locus (3′ integration junction). (**b**) FISH analyses in gene-edited FA-A MEF indicating the integration of the therapeutic PGK-hFANCA donor (red spot signal and red arrows) into chromosome 7, (two green signals corresponding to the two chromatids indicated with a green arrow) where the *Mbs85* locus is located. Co-localization of both signals is indicated with an orange circle. DAPI chromosome staining is shown in blue. Scale bar represents 10 μm for all the microphotographs. Targeted integration event in clone #7# (left) and targeted and non-targeted integration events in clone #76# (right). (**c**) Western blot analysis of hFANCA expression in the bulk gene-edited FA-A MEFs, as well as in the derived clones. Expression levels were calculated as a fold change with respect to β-ACTIN, used as a loading control, and then normalized against the hFANCA expression of lymphoblastic cells from a healthy human donor (HD LCL). FA LCL: lymphoblastic cells derived from a FA-A patient; FA-A: immortalized non-gene-edited FA-A MEFs; WT: immortalized non-gene-edited WT MEFs; FA-AB: bulk of edited FA-A MEFs (T2.5 + D0.75). Analysed clones: #7# and #76#. d) Survival curves in edited FA-A MEF clones together with untransfected WT,FA-A MEFs and FA-A MEFS transduced with a PGK-*hFANCA*-wPRE* LV (FANCA-LV) at MOI 1, after their exposure to MMC (0, 3, 10, 30, 100 nM). Star (★) indicates that no colonies were generated in this condition at a dose of MMC. The survival shown would correspond to the growth of a single colony in these cultures. ^(**)^P-value < 0.01, ^(***)^P-value < 0.001, ^(****)^P-value < 0.0001 indicate significant differences with respect to FA-A group, with a two-way ANOVA followed by a post-hoc Bonferroni test. Asterisks indicate groups with differences (* WT MEFs; ♦ Clone #76#; ● FA-A MEFS + FANCA_LV) with respect to FA-A group. (**e**) Chromosomal aberrations per chromosome induced by MMC, analysed in metaphases of gene-edited FA-A MEF clones, in comparison with non-edited WT and FA-A MEFs.

Seamless integration of the donor into the *Mbs85* site was validated via Sanger sequencing of the PCR amplicon obtained from the 3′ integration junction analysis in one of the five selected clones (Clone #76#, Fig. 3a). Furthermore, metaphase chromosomes were used to characterize the specific PGK-h*FANCA* transgene integration in chromosome 7 (where the *Mbs85* locus is located) by fluorescence *in situ* hybridization (FISH). We used one probe that recognized the two chromatids of chromosome 7 (which appear as two green spots pointed out by a green arrow in Fig. 3b), and a second probe that recognized the PGK-h*FANCA* transgene (which appears as a red spot pointed out by a red arrow in Fig. 3b). Notably, in the two clones analyzed (i.e. #7# and #76#), the red and green signals co-localized in the same metaphase spread, strongly suggesting that the targeted integration correctly occurred at the *Mbs85* locus (Fig. 3b). However, non-targeted integration events were also observed in some of the metaphase spreads, such as in clone #76# (Fig. 3b).

To assure the generation of functionally corrected gene-edited FA-A MEF clones we sought to verify whether these clones were functionally corrected in comparison with their parental non-edited FA-A MEFs. First, the functionality of the cassette was tested to determine the expression of hFANCA by Western blot in gene-edited clones. Since mouse MEFs do not express the human FANCA protein, we normalized the expression of hFANCA using a healthy human donor lymphoblastic cell line (HD LCL). As shown in Fig. 3c, FA-A MEFs, WT MEFs and human FA-A LCLs did not show detectable levels of the hFANCA protein, as expected. However, we observed hFANCA expression both in the bulk population of gene-edited FA-A MEFs (2.5 µg of each TALEN monomer and 0.75 µg of donor) and in clones #7# and #76#. This result demonstrates that our gene-editing approach promoted the expression of the therapeutic h*FANCA* gene in FA-A MEFs upon integration of its expression cassette in the murine *safe harbor Mbs85* locus. As FA cells are characterized by their hypersensitivity to DNA interstrand cross-linking agents, such as mitomycin C (MMC), the sensitivity of uncorrected and gene-edited FA-A MEFs to this drug was tested. Cells were cultured with different doses of MMC and their survival was analyzed at 14 days post-treatment. Selected gene-edited clones #7# and #76# showed increased MMC resistance as compared to non-gene-edited FA-A MEFs (Fig. 3d).

The generation of chromosomal breaks upon exposure to DNA cross-linking drugs is another characteristic of FA cells. Thus, to demonstrate the reversion of the characteristic phenotype of FA cells, chromosomal aberrations in edited FA-A MEF clones were studied in the presence and the absence of MMC. Although the chromosomal instability present in the edited MEFs not exposed to MMC was still higher than that of the FA-A MEFs, possibly due to the replicative stress to which these cells had been exposed, consistent with their restored FA pathway, gene-edited FA-A MEF clones presented a lower number of MMC-induced aberrations per chromosome in comparison with non-corrected FA-A MEFs. Interestingly, the number of chromosomal aberrations per MMC-treated cell was 10.9–12.1 times lower in edited cells as compared to values obtained in their parental non-edited FA-A MEFs (Fig. 3e). Altogether these results demonstrate that the specific integration of the therapeutic PGK-h*FANCA* cassette into the murine *safe harbor Mbs85* locus corrects the disease phenotype of FA-A MEFs.

### Efficient HDR-mediated gene editing in the mouse *Mbs85* locus of WT HPCs

Our laboratory has previously established an efficient and specific gene editing approach to correct fibroblasts and CD34^+^ cells from FA-A patients by harnessing the homology directed repair (HDR) pathway to integrate a therapeutic cassette into the human *AAVS1 safe harbor* locus of these cells^25,26^. Experiments shown in Figs. 1–3 have shown the feasibility of targeting the mouse ortholog of the human *AAVS1 gene* in mouse FA-A fibroblasts. In the subsequent experiments our goal was to test in mouse HSPCs the therapeutic relevance of the gene editing strategy described above to insert the human *FANCA* donor cassette into the murine *AAVS1* ortholog.

**Figure 4:**
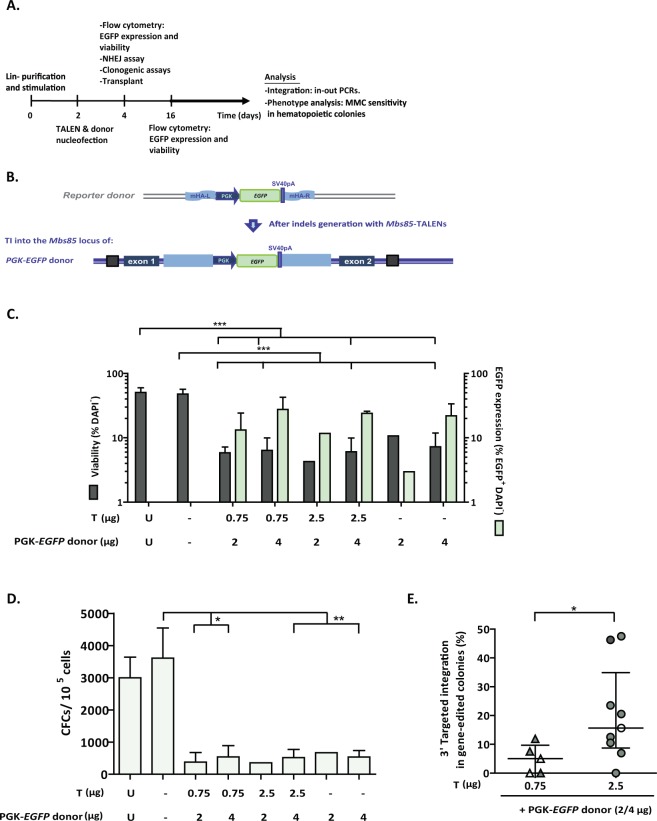
Gene-targeting in WT murine HSPCs. (**a**) Flow chart indicating the study design and the different analyses performed in gene-edited murine HSPCs. (**b**) Schematic showing the architecture of the murine *Mbs85* locus, with the target sites of the TALENs highlighted, and the structure of the donor used with the *EGFP* reporter cassette driven by the phosphoglycerate kinase promoter (PGK) flanked by sequences homologous to the genomic target locus. The resulting locus upon targeted integration (TI) of the donor is indicated in the lowest part of the panel. mHA-L and mHA-R: homology arms to the murine *Mbs85* locus; SV40pA: simian virus 40 polyA sequence; (**c**) Analysis of viability (percentage of DAPI^−^ cells) and percentage of EGFP^+^ cells in WT Lin^−^ BM cells nucleofected with the TALEN and the PGK-*EGFP* reporter donor at 48 hours post-nucleofection. U: untransfected cells; Double negative (-): Nucleofected without DNA; T: different doses of TALEN monomers (from 0.75 to 2.5 µg); D: 2 or 4 µg of the PGK-*EGFP* reporter donor. Data are the mean ± S.D. (n = 5 experiments). ^(***)^P-value < 0.001 indicates significant differences with a one-way ANOVA followed by a *post-hoc* Tukey test. d) Clonogenic assay to evaluate the ability of WT Lin^−^ BM cells to generate hematopoietic colonies under the conditions shown in C). Data are the mean ± S.D. (n = 5 experiments). ^(*)^P-value < 0.05, ^(**)^P-value < 0.01 indicate significant differences with a one-way ANOVA followed by a *post-hoc* Tukey test. (**e**) Targeted integration percentage of the PGK-*EGFP* reporter donor into the *Mbs85* locus of WT Lin^−^ BM cells for the 3′ integration junction calculated in the hematopoietic colonies that were positive for the PCR. Percentages calculated in nucleofected cells with different doses of TALEN (with triangles, 0.75 µg of each monomer; with circles, 2.5 µg of each monomer) and the donor (in white, the dose of 2 µg; in black, the dose of 4 µg). Data are the median ± interquartile range (n = 5–9). ^(*)^P-value < 0.05 indicates significant differences with a Mann-Whitney test.

Lineage negative bone marrow (Lin^-^ BM) cells were purified by cell sorting and then pre-stimulated with a cocktail of hematopoietic cytokines^44,45^ for 48 hours to promote cell cycling of quiescent HSPCs. Upon nucleofection with the TALEN and the therapeutic donor described above, pre-stimulated cells were maintained in culture in the presence of hematopoietic growth factors to boost HDR-mediated gene-editing repair^46^ until performing the analyses at 48 hours post-nucleofection (Fig. 4a).

First, the activity of the *Mbs85*-specific TALENs was evaluated by the Surveyor assay in BM-derived Lin^−^ cells from WT and FA-A mice (Supplementary Fig. 5a**)**. The cleavage efficacies ranged from 30.4% ± 5.9% to 20.5% ± 10.1% respectively, indicating successful disruption of the target locus in HSPCs from these genotypes (Supplementary Fig. 5b). Since HSPCs are more sensitive to *in vitro* manipulation than adherent MEFs and because limited data are available on effective procedures to nucleofect murine HSPCs^47^, we analysed the survival and transfection efficiency of mouse Lin^−^ WT BM cells 48 hours after nucleofection with different doses of TALEN plasmids (0.75 µg or 2.5 µg each monomer) and a PGK-*EGFP* reporter donor (2 µg or 4 µg, respectively), flanked by sequences homologous to the murine *Mbs85* locus (Fig. 4b). A marked reduction (about 7-fold) in the viability of DNA nucleofected samples was observed with respect to untransfected or to mock transfected cells (Fig. 4c).

When cells were nucleofected with different doses of TALEN and PGK-*EGFP* reporter donor, the percentage of EGFP^+^ cells at 48 hours post-nucleofection ranged from 11.7% to 27.6% (transient expression), regardless of the TALENs dose. In the absence of the TALENs, transient expression of EGFP in these cells ranged between 3% and 22% (Fig. 4c). The ability of nucleofected WT Lin^−^ BM cells to generate hematopoietic colonies was also evaluated. DNA nucleofection reduced the ability of BM hematopoietic progenitors to generate colonies approximately 9-fold with respect to mock transfected cells (Fig. 4d).

Although no EGFP-fluorescent colonies were observed in these cultures, we investigated the occurrence of specific integrations of the PGK-EGFP reporter donor into the *Mbs85* locus, by conducting PCRs for the 3′ integration junction in hematopoietic colonies. Strikingly, targeted integration (TI) frequencies of 4.8 ± 2.2% were measured in samples nucleofected with 0.75 µg of each TALEN monomer together with the PGK-EGFP donor, and this value increased to 20.9 ± 9.4% when the TALEN dose was increased to 2.5 µg (Fig. 4e).

### Phenotypic correction of gene-edited FA-A HPCs

To prove the therapeutic potential of gene editing in FA-A HSPCs, the *EGFP* reporter donor used in WT cells was replaced with the therapeutic PGK-h*FANCA* donor used in the MEF experiments (Figs. 1 and 3). Since higher TI frequencies were obtained in WT Lin^−^ cells using 2.5 µg of each TALEN monomer, we used this dose in subsequent experiments, together with 2 and 4 µg of the PGK-FANCA donor.

**Figure 5:**
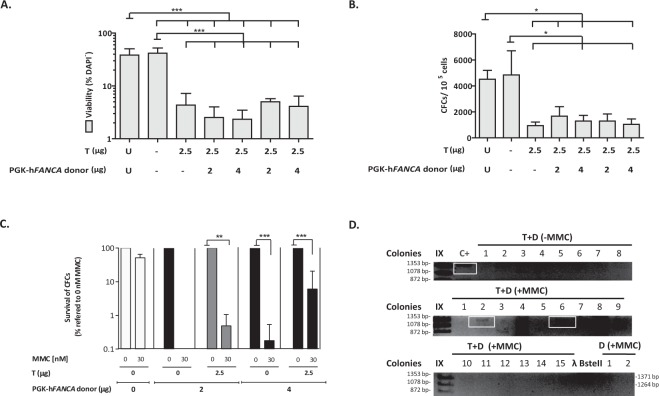
Gene-targeting in FA-A murine HSPCs. (**a**) Analysis of viability (percentage of DAPI^−^ cells) in FA-A Lin^−^ BM cells nucleofected with the TALEN and the therapeutic PGK-h*FANCA* donor at 48 hours post-nucleofection. U: untransfected cells; Double negative (-): Nucleofected without DNA; T: 2.5 µg of each NN-TALEN monomer; D: 2 or 4 µg of the therapeutic PGK-h*FANCA* donor. Data are the mean ± S.D. (n = 7 experiments). ^(***)^P-value <0.001 indicates significant differences with a one-way ANOVA followed by a *post-hoc* Tukey test. (**b**) Clonogenic assays of nucleofected Lin^−^ cells under the conditions shown in A). Data are the mean ± S.D. (n = 7 experiments). ^(*)^P-value < 0.05 indicate significant differences with a one-way ANOVA followed by a *post-hoc* Tukey test. (**c**) Phenotypic correction measured with clonogenic assays in nucleofected FA-A Lin^−^ BM cells treated with 30 nM MMC compared to cells cultured in the absence of MMC and compared with historical data of resistance to MMC in WT mice. MMC survivals are indicated considering the number of hematopoietic colonies generated without drug selection as 100%. Cells were subjected to nucleofection with 2.5 µg of each TALEN monomer and the therapeutic PGK-h*FANCA* donor (2 and 4 µg). Data are presented as mean ± S.D. (n = 4–7 with the except of D2 µg where n = 2 experiments). No statistical differences were found among groups with a non-parametric Kruskal-Wallis and median test. d) Representative PCR analysis for the study of the 3′ integration junction (1,337 bp). C+: sequenced genomic DNA positive for the integration junction in edited FA-A MEFs; T + D: samples from nucleofected FA Lin^−^ BM cells with 2.5 μg of each TALEN monomer together with 4 μg of the therapeutic PGK-h*FANCA* donor; D: 4 μg of the therapeutic PGK-h*FANCA* donor; IX and λ BstII: DNA molecular weight markers. Analysed colonies are numbered and the positive ones framed in blue.

Nucleofection of FA-A Lin- BM cells with the TALEN and the donor plasmids reduced the viability of these cells 11-fold either when compared with non-nucleofected or mock nucleofected cells (Fig. 5a). Similarly, DNA nucleofection also reduced the clonogenic ability of FA-A Lin^−^ BM cells approximately 9-fold in comparison with cells subjected to nucleofection in the absence of DNA (Fig. 5b), which is similar to what we observed in nucleofected WT Lin^−^ BM cells (no significant differences were observed using a two-way ANOVA).

Remarkably, when cells were plated in methylcellulose in the presence or absence of 30 nM MMC, 6% of the hematopoietic colonies corresponding to samples nucleofected with the TALEN and the therapeutic donor survived in the presence of MMC, while only 0.2% of colonies survived in MMC when only the donor was used, indicating that targeting the integration of the therapeutic transgene to *Mbs85* corrected the hypersensitivity of FA HSPCs to MMC (Fig. 5c). In 16.3% of the colonies that grew in the presence of MMC we confirmed the amplification of the expected Mbs85 3´ integration junction (Fig. 5d), thereby indicating that correction of MMC-hypersensitivity in primary FA-A mHSPCs was a consequence of the PGK-h*FANCA* targeted integration in the *Mbs85* locus.

## Discussion

The *AAVS1* locus has been defined as a bona-fide *safe harbor* locus in humans^34^, in which an exogenous gene could be efficiently expressed in various cell lines and iPSCs^28–34,48–52^. Therefore, in this work, we have decided to carry out a targeted integration strategy into the mouse *Mbs85* orthologous locus in order to asses if the corresponding mouse locus may serve as a new site where gene editing can be performed in mouse disease models such as Fanconi anemia (FA). In the future, a deeper and comprehensive characterization of the locus would be desirable, in order to evaluate, in a better meaningful manner, the resemblance or the extent of this *safe harbor* locus for *in vitro* and *in vivo* systems, with variables such as homology–dependent and –independent integration; cell type-specific gene transfer efficiencies; repair template type (single- vs double –stranded); length and degree of nucleotide sequence identity between the repair template and the target homology arms; a comparative transgene integration efficiency analysis versus other well-stablished mouse locus such as *Rosa26*; and expression and functionality of the endogenous *Mbs85* locus.

Gene editing of HSPCs is an attractive strategy in FA due to the *in vivo* proliferation advantage of corrected HSPCs over non-corrected ones^53–59^. TALEN delivery has remained one of the major obstacles in providing short-term and dose-controllable nuclease activity based on previous studies^60–63^. We nucleofected a pair of TALENs in combination with a homologous donor template, in the form of plasmid DNA, in both MEFs and HSPCs from wt or FA-A mice. Carrying out targeted integration in FA-A cells constitute challenging approaches of gene therapy due to the involvement of the FA proteins in promoting HDR^64,65^, which could affect the efficiency of the homologous integration of the therapeutic cassette in the targeting site of the genome. Nonetheless, while this FA-A mouse model is the only disease-relevant model for FANCA deficiency^66^, it does not fully recapitulate the human condition as it presents a mild phenotype. And even if this phenotype could be rescued by transduction of the hematopoietic progenitors with a vector expressing the human FANCA cDNA^13,67^, this mild phenotype could be behind our observations of gene editing efficacy and feasibility using FA mouse cells, in comparison with studies of gene editing that have been done in our laboratory using FA-A human cells that had demonstrated higher targeting efficiencies^25,26^.

Once the expression of TALEN monomers in HEK293T cells was confirmed (Supplementary Fig. 1b), we established proof of principle for gene editing in FA-A MEFs. Using a stepwise experimental approach as followed in previous gene-targeting studies^25,68,69^, we observed relatively high transfection efficiencies (mean value of 30%) and viabilities (ranging from 47% to 78%), indicating that in this cell type cytotoxicity was not a limiting factor to perform gene editing. We also observed that when a donor template was used together with the TALENs, the percentage of repair by NHEJ was significantly reduced; suggesting that a proportion of NHEJ repair was replaced by HDR in the presence of a donor template.

A thorough analysis of the off-target effects is crucial for the further development of gene-editing based therapeutics. We used high-throughput sequencing to assess the off-target sites previously predicted *in silico* with the PROGNOS software^43^ in nucleofected MEFs, as this method is the preferred one to detect indel mutations induced at low frequencies with great sensitivity^70,71^. Indels generated by the nucleases were highly specific in WT and FA-A MEFs, as demonstrated by the high percentage of on-target indels and the low percentage of off-target generated indels (Supplementary Table 1). As previously shown for other TALEN^42,72^, our study confirms the high specificity of the *Mbs85*-specific TALENs.

In some colonies, we had difficulties amplifying the specific PCR bands corresponding for both integration junctions. This could be due to the efficiency of the PCRs, or to the limited and poor quality of the DNA obtained from single colonies. However, as has been previously mentioned, other mechanisms of repair of the DSBs could have been involved apart from HRR, giving rise to different outcomes at the different junctions. Importantly, most of the colonies that arose from samples only nucleofected with the donor did not amplify any of the specific integration bands. In the few cases in which this was observed, on-target non-directed integration events independent of TALEN cleavage may have occurred. Considering that in FA, genetic instability is a fact that could also increases the chance of somatic reversion of its constitutional mutation, in the future, new approaches intended to study the on-target integration with a specific donor but without the need of nuclease delivery would be interesting to explore, specially using cells with the FA-A disease phenotype, as if there is a selective advantage for a small number of cells corrected by this way, this would be a strategy very attractive in the field.

We confirmed specific integration of the therapeutic PGK-h*FANCA* cassette into the *Mbs85* locus by PCR when both the donor and the TALENs were nucleofected simultaneously, indicating the generation of HDR-mediated integrations of the therapeutic donor at the on-target site (Fig. 1e). Importantly, the specific integration was confirmed by Sanger sequencing (Fig. 3a) and FISH (Fig. 3b). Notably, although we detected a large polyploidy, typical of MEFs that have been exposed to cell culture and immortalization, in the two clones analyzed (i.e. #7# and #76#), the red and green signals co-localized in the same metaphase spread, strongly suggesting that the targeted integration correctly occurred at the *Mbs85* locus (Fig. 3b). However, we also observed the occurrence of non-targeted integration events, highlighting the risks associated with DNA delivery (Fig. 3b, clone #76#).

In this study, we also confirmed the efficient phenotypic correction of edited MEF cells. This conclusion was deduced both from the functional expression of the hFANCA protein (Fig. 3c) and the MMC resistance (Fig. 3e) and reduced MMC-induced chromosomal instability (Fig. 3f) of edited clones. Taken together, these studies demonstrate the correction of the FA/BRCA pathway through TALEN-mediated targeted integration of a therapeutic h*FANCA* cassette into the *Mbs85* locus of FA-A MEFs.

Once we proved the efficacy of our gene-targeting experiments in FA-A MEFs, we performed similar experiments in mouse HSPCs in order to demonstrate the feasibility of a gene-targeting strategy in the *Mbs85* locus of these cells using the PGK-*EGFP* reporter donor. Working with sorted Lin^−^ BM cells from WT mice, we observed the expression of the integrated *EGFP* (Supplementary Fig. 6), facilitated by the targeted integration of the *EGFP* cassette into the *Mbs85* locus. Despite the absence of EGFP expression in *Mbs85-*edited WT HSPCs, targeted integration in up to 20.98 ± 9.4% of clones were observed using 2.5 µg of each TALEN monomer together with 4 µg of the *EGFP* reporter donor (Fig. 4e). This observation and the very low proportion of EGFP-expressing cells observed in liquid cultures suggests a restricted expression of transgenes in the *Mbs85* locus of mHSPCs as has previously been reported in the human *AAVS1* locus of hESCs^51^.

Consistent with the results observed in WT Lin^−^ BM cells, we demonstrated for the first time the feasibility of conducting a therapeutic gene targeting approach into the *Mbs85* locus of primary Lin^−^ BM cells from FA-A mice using the PGK-h*FANCA* donor. As for WT mHSCs, both the viability and clonogenic potential of nucleofected FA-A mHSCs (Fig. 5a,b) declined sharply. Regarding the targeted integration observed in WT as compared to FA HSPCs, similar efficacies were observed, supporting the hypothesis that although HDR could be moderately affected in FA-A cells (as was observed in a recent study from our laboratory^25^), gene editing is feasible in these cells, due to their mild HDR defects, in comparison with FA-D1 cells.

Of particular importance is the fact that our results show the correction of the MMC-hypersensitivity phenotype in primary FA-A mHPCs. Correctly edited FA-A HPCs survived cytotoxic concentrations of MMC, similarly to what was observed in FA-A MEFs, highlighting the therapeutic potential of the proposed gene therapy approach. Of particular importance is the fact that, the low level of correction without the presence of TALEN (0% and 0.18% respectively depending of the concentration of donor, 2 or 4 µg) shown in mHPCs in Fig. 5c clearly point out that the percentage of off-target integration is derisory once you select the cells with an antibiotic such as MMC. Moreover, the ratio of targeted to random integration in classic gene targeting approaches is very low, because HRR depends on the homologous arms of the donor invading the intact double strand of the corresponding counterparts in the genomic DNA. Moreover, the use of designer nucleases strongly increases the probability of HRR reducing that of random integration. As a result, the ratio of correctly targeted clones versus clones with random integration is substantially shifted towards targeted integration although negative selection increases this percentage, as it was our case with FA-A MEFs (with puromycin) and HPCs (with MMC). Although *Mbs85* might limit the efficacy of expression of integrated cassettes in this locus and should be further study, we have demonstrated the achievement of significant levels of MMC-resistant in edited FA-A CFCs, which is consistent with our previous observations showing that low levels of FANCA can result in a therapeutic effect^12,73^.

In our studies of DNA nucleofection in mouse HSPCs, we observed a marked cytotoxicity due to transferring plasmid DNA to these cells, as mock nucleofection showed no impairment in viability (Fig. 4d,e). We have not been able to demonstrate the engraftment of e*x vivo* gene edited HSPC into syngeneic recipient mice due to the high toxicity observed in FA-HSPCs that provided so low number of cells for the transplant. This toxicity that results in a reduction in the efficiency of these strategies, currently constitutes one of the main limitations to conduct *ex vivo* therapeutic approaches of gene editing in hematopoietic diseases^74^, however, other delivery methods might be safer and overcome this issue^25,75–79^. The optimization of different strategies including RNA delivery has been studied throughout this work, although they have not been shown in this manuscript. However, the shortage of delivery materials compatible with mouse Lin^−^ cells has demonstrate these the best results we have obtained in our hands. Nevertheless, our results showing toxicity of gene editing approaches in mouse HPCs are consistent with previous studies aiming at the gene targeting in primary mHPCs^45,47,80^ and confirm that gene editing in the murine HSPC compartment is challenging, particularly in cells capable of hematopoietic repopulation.

Overall, our data provide evidence of successful therapeutic gene editing with TALE nucleases, into the mouse *Mbs85* orthologous locus in fibroblasts and HPCs of a mouse model of FA-A, and establishes the rationale and the proof of principle to use this locus for the gene correction in other diseases to investigate the efficacy and safety of future gene editing strategies.

## Methods

### Cells and cell culture

HEK-293T (ATCC-CRL-3216™) cells were cultured in DMEM 1X with GlutaMAX^TM^ (Gibco, Life Technologies/Thermo Fisher) with 10% Hyclone (GE Healthcare) and 1% Penicillin/Streptomycin (P/S) (Gibco).

MEFs both from FVB/NJ WT and FVB FA-A mice were obtained from the chorion of 13.5 E pregnant females^81^. MEFs from WT or FA-A mice were immortalized by a transient transfection with pLXSN 16 *E6E7* and pCL-ECO-gag-pol^82^ using the CaCl_2_ DNA precipitation method and cultured also in DMEM 1X with GlutaMAX^TM^ (Gibco) with 10% Hyclone (GE Healthcare) and 1% P/S (Gibco).

BM cells were isolated from FVB/NJ WT, FVB FA-A or C57BL/6J mice by flushing the femurs and tibias of these mice in IMDM (Gibco). The cellular suspension was incubated with lysis solution (CINH_4_ with CO_3_HK 1 M with EDTA 0.5 M) (Merck KGaA) for 5 minutes at RT in darkness. Then, cells were washed with PBS 1 × (Sigma® Life Sciences) with 5% Hyclone and 5% P/S. Purified mouse hematopoietic progenitor cells (HPCs), Lin^−^ (lineage-negative) cells, were obtained by whole BM cell sorting by immunoselection. Lin^−^ were expanded in StemSpan™ (StemCell™ Technologies) with 1% GlutaMAX™ with cytokines and 1% P/S. The following cytokines were added: 100 ng/ml mouse stem cell factor (mSCF), 100 ng/ml human interleukin 11 (hIL-11), 100 ng/ml human FMS-like tyrosine kinase 3 ligand (hFlt3), 100 ng/ml human thrombopoietin (hTPO)(EuroBioSciences).

With the exception of HEK-293T cells that were cultured at standard normoxic (21% O_2_–5% CO_2_) conditions the rest of the cells were cultured in hypoxia (5% O_2_-5% CO_2_) at 37 °C, and 95% relative humidity.

Animals were maintained at the Animal Facility Laboratory of CIEMAT (registration number ES280790000183). All experimental protocols and methods used were performed in accordance with the guidance and approval of the corresponding Spanish regulations regarding experimental animal welfare (RD 223/1998 and Directive 2010/63/EU protocols). The experimental protocol was reviewed and approved by the ethics committee for animal research of the OH-OEBA CIEMAT (“Organo encargado del Bienestar Animal del Centro de Investigaciones Energeticas y Medioambientales” by the resolution 10/085231.9/14); (Proex. 070/15) and the “Consejeria de Medioambiente y de Ordenación del Territorio” from the Comunity of Madrid (based on the RD 53/2013).

### Transcription activator-like effector nuclease (TALEN), donors and control plasmids

TALE-based DNA binding domains were assembled using Golden Gate assembly kit^83^ modified based on our previously optimized TALEN scaffold^42^ (Δ135/+ 17). The vectors used^72^ included the 17.5th repeat and the wild-type *Fok*I cleavage domain (pVAX_CMV_TALshuttle(xx); ‘xx’ stands for the four different 17.5th RVDs used, NI, NG, HD and NN).

Donor plasmids were flanked by two homology arms (HA) of the *Mbs85* locus of 806 bp and 860 bp, respectively. The therapeutic donor contained a PGK.FANCA.E2A.PuroR.SV40pA fragment that was chemically synthesized by GenScript. PGK-*EGFP* reporter donor was cloned in the backbone of the therapeutic cassette by digestion of the PGK.*EGFP* fragment from the pCCL.PGK.*EGFP*.wPRE* plasmid with EcoRI/NotI restriction enzymes (New England Biolabs). A PGK-h*FANCA* donor with longer homology arms, was used as a positive control for targeted integration in the *Mbs85* locus.

### Lipofection and nucleofection

HEK-293T cells were lipofected one day after seeding 1 × 10^5^ cells with Lipofectamine® 2000 Reagent according to manufacturer’s protocol (Invitrogen, Life Technologies, Thermo Fisher Scientific). 400 ng of DNA of each TALEN monomer were co-transfected with 100 ng of an *EGFP* control plasmid and 500 ng of pUC118 control plasmid.

For nucleofection of WT or FA-A immortalized MEFs, 2 × 10^6^ cells per condition were used with the Amaxa MEF2 Nucleofector® Kit (Lonza Group) using program T20 of the Nucleofector™ I device, 2.5 µg of each TALEN monomer were nucleofected together with a determined donor dose (0.75, 2 and 4 µg). Enrichment during 5 passages with puromycin (1–1.25 µg/ml) was performed, then clones were generated by limiting dilution to perform gene targeting studies.

For the BM hematopoietic cells, 1.4 × 10^6^ cells of purified BM Lin^−^ cells were nucleofected with an *EGFP* control plasmid or the corresponding doses of the TALEN monomers and donors after pre-stimulation during 48 h. Lin^−^ BM cells were nucleofected with the 4-D Nucleofector™ device using the P3 Primary Cell 4D-Nucleofector® X Kit (Lonza Group) kit with the ED-113 program.

### Cell sorting & flow cytometry

Lin^−^ cells were purified from C57BL/6J and FA-A FVB/NJ mice by cell sorting using lineage-specific antibodies phycoerytrin conjugated (BD Pharmingen) (anti-B220 (CD45R), anti-Mac-1 (CD11b), anti-Gr1 (Ly6G/C), anti-CD3-ε, and anti-Tert-119 antibodies) at a concentration of 0.06 μg/mL and 0.02 μg/mL, respectively. For the identification of LSK cells (Lineage negative, Sca-1^+^, c-Kit^+^), Lin^−^ cells were stained with the described cocktail, and with 0.06 μg/mL of anti-Sca-1-APC-Cy7 (BioLegend) and 0.15 μg/mL of anti-c-Kit-A647 antibodies (Southern). 4′,6-Diamidino-2-phenylindole (DAPI; Roche)-negative staining was used as a marker of cell viability. Analyses were performed in the LSR Fortessa cell analyser (BD). Transfection efficiency was also determined by FACS analysis 48 h post nucleofection. Off-line analyses were conducted with the FlowJo Software v7.6.5 (© FlowJo, LLC).

### Clonogenic -colony forming cell (CFC)- assays of hematopoietic progenitors and colony forming units (CFUs)

CFC assays were performed following manufacturer´s recommendations (Stem Cell Technologies). MMC (at 10 and 30 nM) (Sigma® Life Sciences) and puromycin (1 µg/ml) (Sigma® Life Sciences) was added, respectively, to analyze the number of gene-edited cells. 2 × 10^5^ cells were seeded at 48 hours post-nucleofection. Colonies were scored in an inverted microscope (Olympus IX70 WH10X/22). Cultures were maintained in hypoxia at 37 °C, and 95% relative humidity.

Survival of immortalized and targeted FA-MEFs was analyzed by scoring the number of colonies derived from 200 cells (CFUs, colony forming units) exposed to MMC (0, 3, 10, 30, 100 and 300 nM). After one week, the medium was changed with fresh medium containing the same concentration of MMC. Cell viability was determined 14 days after cell seeding.

### *Cel I* (Surveyor) analysis of TALENs pairs activity

DNA was extracted using NucleoSpin® Tissue kit (Macherey-Nagel) from cellular pellets obtained at 48 h or after cell expansion. PCR fragments spanning the *Mbs85* TALENs target site were generated with primers mAAVS1 CelIF and mAAVS1 CelIR (Supplementary Table 4). PCR was performed as follows: 200 ng of gDNA, 1.25 µl 10 µM of each primer, 0.5 µl 100 nM dNTPs, 10 µl of the Buffer 10X and 1 µl of Herculase enzyme (Herculase II Fusion Enzyme) in a final volume of 50 µl. Cycling conditions were the following: 95 °C for 2 minutes, 40 cycles of 95 °C for 20 seconds, 56 °C for 20 seconds and 72 °C for 30 seconds, and finally one cycle of 72 °C for 3 minutes. PCR products were purified with the NucleoSpin® Gel and PCR Clean-up kit (Macherey Nagel) and amplified following the manufacturer’s instructions. Final products were migrated and revealed according to manufacturer instructions and analyzed in a Molecular Imager® GelDoc™ XR + System (BioRad). Quantification of the bands was performed using the Quantity One Software (BioRad).

### On-target integration analyses

Genomic DNA from either the bulk cell population or from FA-A MEF clones was extracted using NucleoSpin® Tissue kit (Macherey Nagel). gDNA from single colonies derived from CFCs assays was extracted as previously described^84^. Two PCR reactions were conducted both for the 5′ and the 3′ integration junctions of the *Mbs85* integration site. To analyze the integration of the PGK-h*FANCA* donor the following primers were used: the pair mAAVS1-5′F (1 & 2) and mAAVS1-5′R (1 & 2); and the pair mAAVS1-3′F (1 & 2) and mAAVS1-3′R (1 & 2) (Supplementary Table 4). To analyze the integration of the PGK*-EGFP* donor: the pair mAAVS1-5′F3 and mAAVS1-5′R3, and the pair mAAVS1-EGFP-3′F and mAAVS1-EGFP-3′R were used. PCR reactions were conducted using 200 ng of gDNA from bulk cell population or single MEF clones, or 15 µl of gDNA from single colonies. PCRs from the 5′ and the 3′ integration junctions were performed with 1.25 µl at 10 µM of each primer, 0.5 µl at 100 nM dNTPs, 10 µl of the Buffer 10X and 1 µl of Herculase enzyme in a final volume of 50 µl. Cycling conditions were the following: 95 °C for 10 minutes, 40 cycles of 95 °C for 30 seconds, 59 °C -mAAVS1-5′F1&R1-; or 58 °C -mAAVS1-5′F2&R2-; or 62 °C -mAAVS1-5′F3&R3- for 60 seconds (for the 5′ integration junction depending on the primers used); or 59 °C -mAAVS1-3′F1&R1-; 61 °C -mAAVS1-3′F2&R2-; 62 °C -mAAVS1-EGFP-3′F&R- for 60 seconds (for the 3′ integration junction depending on the primers used); and 72 °C for 1.5 minutes; and finally one cycle of 72 °C for 10 minutes in a final volume of 50 µl.

PCR products of the 3′ integration junction performed in the FA-A MEF bulk population were sequenced by Sanger method. Primers used for sequencing were the followings: mAAVS1-3′-F2, mAAVS1-3′-R2; SeqAAVS1-1_3′_F; SeqAAVS1-2_3′_F; SeqAAVS1-3_3′_F; SeqAAVS1-4_3′_F; SeqAAVS1-1_3′_R (Supplementary Table 4). Analyses of the sequences were done with Finch TV version 1.4.0 and Vector NTI software.

### Fluorescence *in situ* hybridization (FISH)

Cells were first arrested in metaphase with KaryoMAX Colcemid solution (Gibco) and then harvested after a treatment with a hypotonic salt solution. Two sets of probes were used to localize plasmid integration site. RP23-336p21 and RP24-129K5 specific bacterial artificial chromosomes (BACs) that map to the E3 band of the mouse chromosome 7 (Human BAC Clone Library, Children’s Hospital Oakland Research Institute [CHORI]) were used as controls. PGK-h*FANCA* transgene integration site was detected using as probe the DNA from the plasmid vector. Following the manufacturer’s specifications, BACs DNA was directly labelled by nick translation (Vysis, Abbott Molecular) with SpectrumGreen-dUTPs, whereas plasmid DNA was labelled with SpectrumOrange-dUTP (Vysis). The probes were blocked with Cot-1 DNA and DNA sheared salmon sperm (Vysis) to suppress repetitive sequences, and hybridized overnight as 37 °C onto metaphase spreads. After post-hybridization washes, the chromosomes were counterstained with DAPI in Vectashield mounting medium (Vector Laboratories). Cells images were captured using a cooled charge-coupled device (CCD) camera (Photometrics SenSys camera) connected to a computer running a Chromofluor image analysis system (CytoVision, Leica Biosystems).

### Western blot

Western blot (WB) analyses were performed to determine the expression of the HA epitope of TALENs in HEK-293T cells using anti-HA antibody (NB600-363, Novus Biologicals). Human FANCA protein was also analyzed in immortalized FA-A MEFs and in gene-edited FA-A MEFs as previously described^26,85^.

### Chromosomal instability assay

Gene-edited FA-A MEFs were subjected to medium containing 40 nM of MMC for 24 hours to induce DNA damage. Then, 0.05 µg/ml of colcemid (KaryoMAX Colcemid Solution, Gibco) was added to the cells followed by treatment with 0.56% KCl during 15 min at 37 °C and fixed in methanol: acetic acid (3:1). Extensions were made at 25 °C with 48% humidity in a Thermotron chamber. Metaphases were stained with 10% Giemsa stain, giemsa’s azur eosin methylene blue solution (Merck) with Gibco® Gurr Buffer Tablets (Gibco). Then, cells were placed in glass coverslips with a pair of droplets of Entellan (Merck). In general, a minimum of 20 metaphases per culture were studied for the analysis of aberrations. Cell images were taken with an inverted microscope (Olympus CK30).

### Off-target analysis

Possible off-target sites for the TALENs pair targeting the sequences 5′-TGTCCTCTCTTCTTGCTAG and 5′-AGTTACTGGTGGGAACAGA within the mm10 genome were predicted using the online tool PROGNOS^43,86^. In the submitted query, 6 mismatches for each TALEN-half-site and a spacing distance between 10 bp and 30 bp were considered, together with hetero- and homodimeric sites (indicated in Supplementary Table 2). The 24 top ranked off-targets (TALENv2.0 algorithm from PROGNOS) were analyzed by deep sequencing.

WT and FA-A MEFs were either treated with corresponding nucleases or left untreated. Genomic DNA was isolated to PCR amplify all predicted off-target loci (50–100 ng DNA were used per target; primers are listed in Supplementary Table 2). Libraries were prepared from PCR Amplicons using the NEBNext® Ultra™ II DNA Library Prep Kit for Illumina® (NEB, E7645) and quantified using the ddPCR™ Library Quantification Kit for Illumina TruSeq (Biorad, #186-3040). All samples were sequenced on an Illumina MiSeq platform with a MiSeq Reagent Kit v2, 500cycles (Illumina, MS-102-2003).

Paired-end reads from MiSeq reactions were quality trimmed by an average Phred quality (Qscore) greater than 20 using TrimGalor (www.bioinformatics.babraham.ac.uk/projects/trim_galore) and merged into a longer single read with a minimum overlap of 30 nucleotides using Fast Length Adjustment of Short reads (FLASH)^87^. Merged sequences were aligned against all reference sequences (Supplementary Table 2) using Burrows-Wheeler Aligner (BWA). Alignments were analyzed for insertions and deletions within a range of ±20 bp of the predicted nuclease cleavage site.

### Statistical analyses

Statistical analyses were performed using GraphPad Prism version 5.0 for Windows (GraphPad Software). Results are shown as the mean ± Standard Deviation (SD) or as the median ± interquartile range, from at least 3 replicates and from at least 3 experiments (indicated in the footnotes of figures). The mean ± Standard Error of the Mean (S.E.M) has only been used for the calculation of the specific site integration in hematopoietic progenitors. When two sets of data were compared, two-tailed Student’s t-test or Mann-Whitney tests were performed depending on whether or not the values followed a normal distribution. Statistical significance of the indel frequency occurred at predicted off-target sites was determined using a one-tailed, homoscedastic Student’s t-test. This test was performed to compare the values that were above those obtained in untreated cells. When more than two sets of data were compared, a parametric one-way ANOVA followed by post-hoc multiple comparison Tukey test or a non-parametric Kruskal-Wallis followed by a post-hoc Dunn’s multiple comparison test were used. In some experiments, more than one variable was analyzed. In these cases, when the data suited a normal distribution, the two-way ANOVA test was applied followed by a Bonferroni post-hoc test. Significant differences were indicated as ^(*)^P-value < 0.05, ^(**)^P-value < 0.01, ^(***)^P-value < 0.001 and ^(****)^P-value < 0.0001.

## Supplementary information

Supplementary information.
