# Supplementary material for: TALEN mediated gene editing in a mouse model of Fanconi anemia
Source: Sci Rep. 2020 Apr 24;10:6997. doi: 10.1038/s41598-020-63971-z (PMC7181878; doi:10.1038/s41598-020-63971-z)
Supplement: Supplementary file 1 — Supplementary information. [file 41598_2020_63971_MOESM1_ESM.pdf]

TALEN mediated gene editing in a mouse model of Fanconi anemia

Maria José Pino-Barrio[1,2,3], Yari Giménez[1,2,3], Mariela Villanueva[1,2,3], Marcus Hildenbeutel[6,7], Rebeca Sánchez-Dominguez[1,2,3], Sandra Rodríguez-Perales[4], Roser Pujol[3,5], Jordi Surrallés[3,5], Paula Río[1,2,3], Toni Cathomen [6,7,8], Claudio Mussolino[6,7], Juan Antonio Bueren[1,2,3]* & Susana Navarro[1,2,3]*

1Division of Hematopoietic Innovative Therapies, CIEMAT/CIBERER, 28040, Madrid, Spain.
2Advanced Therapies Unit, IIS-Fundación Jimenez Diaz (IIS-FJD, UAM), 28040, Madrid, Spain.
3Center for Biomedical Network Research on Rare Diseases, Instituto de Salud Carlos III, Madrid, Spain.
4Molecular Cytogenetics Group, Human Cancer Genetics Program, Centro Nacional de Investigaciones Oncológicas (CNIO), Melchor Fernandez Almagro, 3, 28029, Madrid, Spain.
5Genome Instability and DNA Repair Group, Department of Genetics and Microbiology, Universitat Autònoma de Barcelona, 08193, Barcelona, Spain.
6Institute for Transfusion Medicine and Gene Therapy, Medical Center - University of Freiburg, 79106, Freiburg, Germany. 7Center for Chronic Immunodeficiency, Medical Center - University of Freiburg, 79106, Freiburg, Germany.
8Faculty of Medicine,University of Freiburg, Freiburg, Germany.

*Corresponding authors:

Susana Navarro, Telephone: +34913460891. Fax: +34913466484. E-mail: s.navarro@ciemat.es
Juan A. Bueren, Telephone: +34913466518. Fax: +34913466484. E-mail: juan.bueren@ciemat.es;

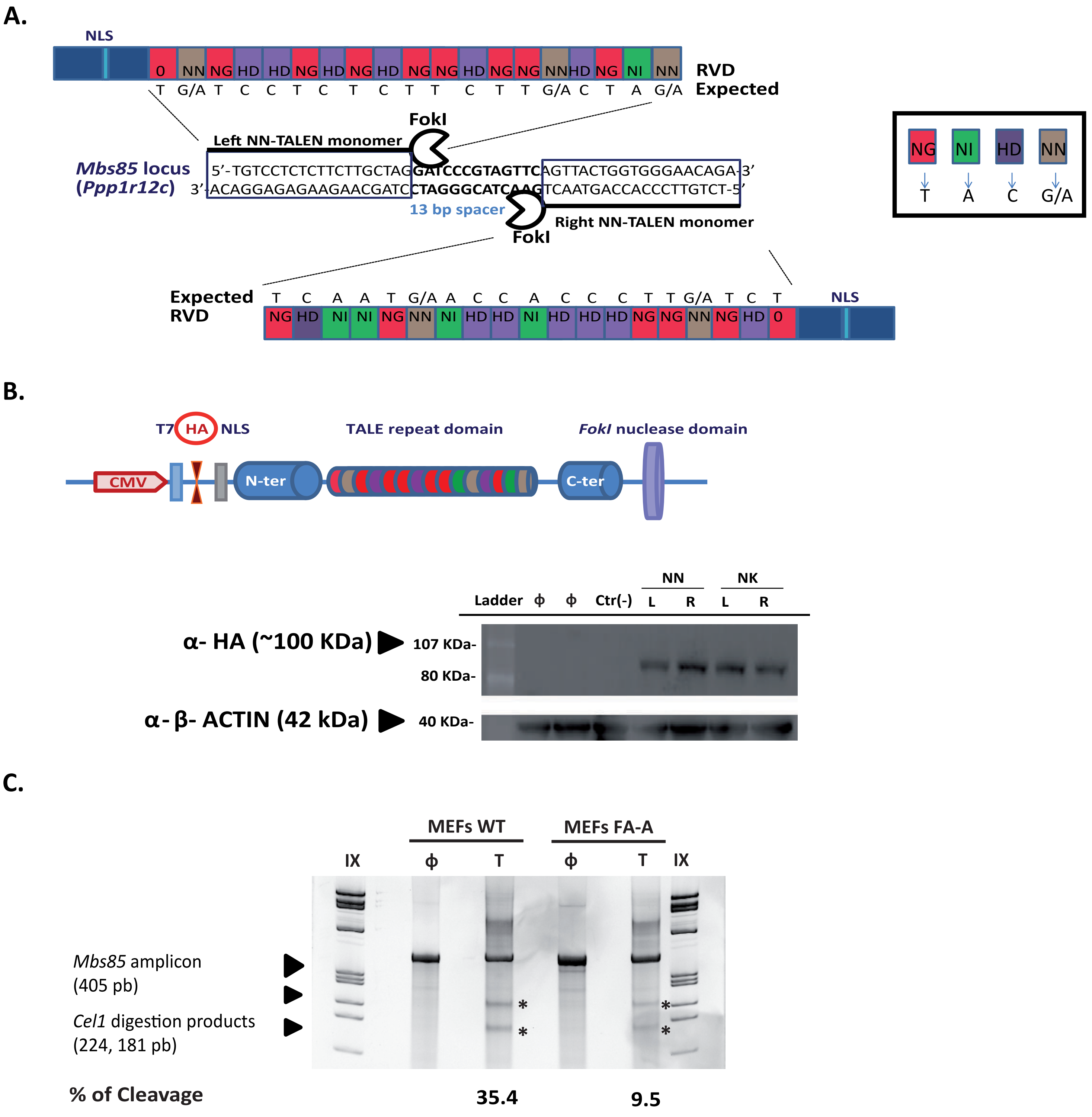

Supplementary Figure 1

a

```
                        215
                         |
222+4: ...GTAACTGAAC  tacg  TACGGGAT...
223+3: ...GTAACTGAACT  acg  ACGGGAT...
223+4: ...GTAACTGAACT  acgg  ACGGGAT...
225+1: ...GTAACTGAACTAC  g  GGGAT...
222+2: ...GTAACTGAAC  ta  TACGGGAT...
226+3: ...GTAACTGAACTACG  tac  GGAT...
225+5: ...GTAACTGAACTAC  ggacg  GGGAT...
225+3: ...GTAACTGAACTAC  gta  GGGAT...
226+4: ...GTAACTGAACTACG  gtac  GGAT...
224+4: ...GTAACTGAACTA  ccgg  CGGGAT...
224+2: ...GTAACTGAACTA  cg  CGGGAT...
222+1: ...GTAACTGAAC  t  TACGGGAT...
220+3: ...GTAACTGA  act  ACTACGGGAT...
219+5: ...GTAACTG  ttact  AACTACGGGAT...
225+1: ...GTAACTGAACTAC  a  GGGAT...
224+2: ...GTAACTGAACTA  gg  CGGGAT...
225+4: ...GTAACTGAACTAC  ggcg  GGGAT...
226+2: ...GTAACTGAACTACG  ac  GGAT...
222+5: ...GTAACTGAAC  ttacg  TACGGGAT...
225+5: ...GTAACTGAACTAC  tacta  GGGAT...
223+2: ...GTAACTGAACT  cg  ACGGGAT...
222+1: ...GTAACTGAAC  a  TACGGGAT...
222+1: ...GTAACTGAAC  g  TACGGGAT...
225+4: ...GTAACTGAACTAC  gacg  GGGAT...
```

b

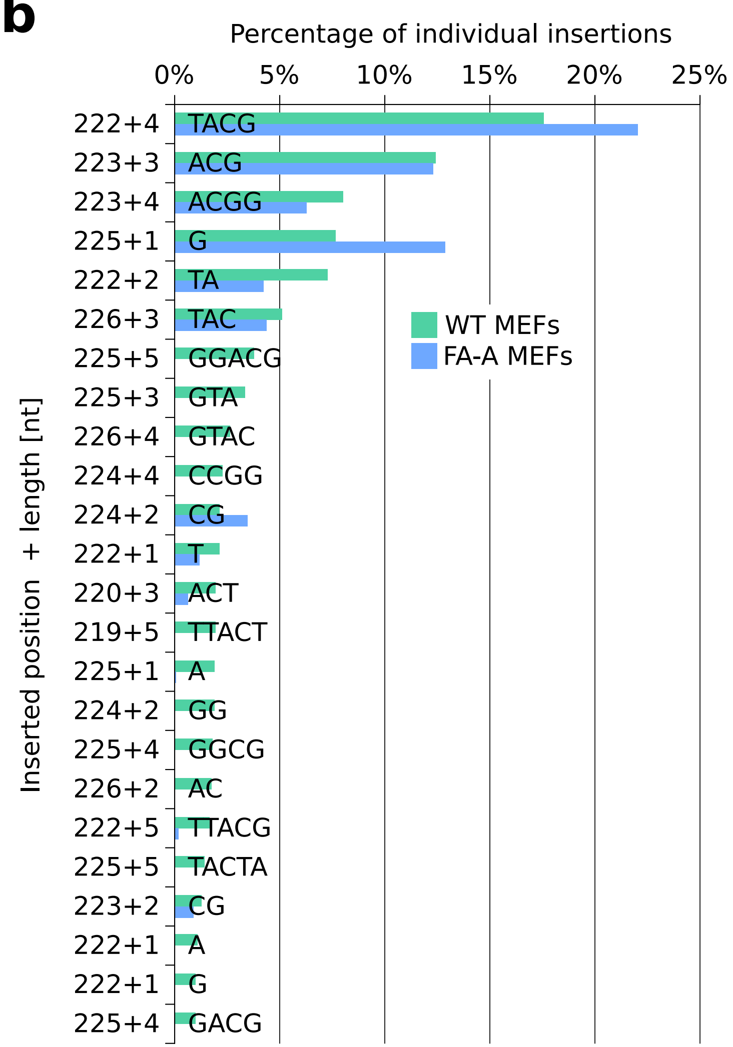

Supplementary Figure 2

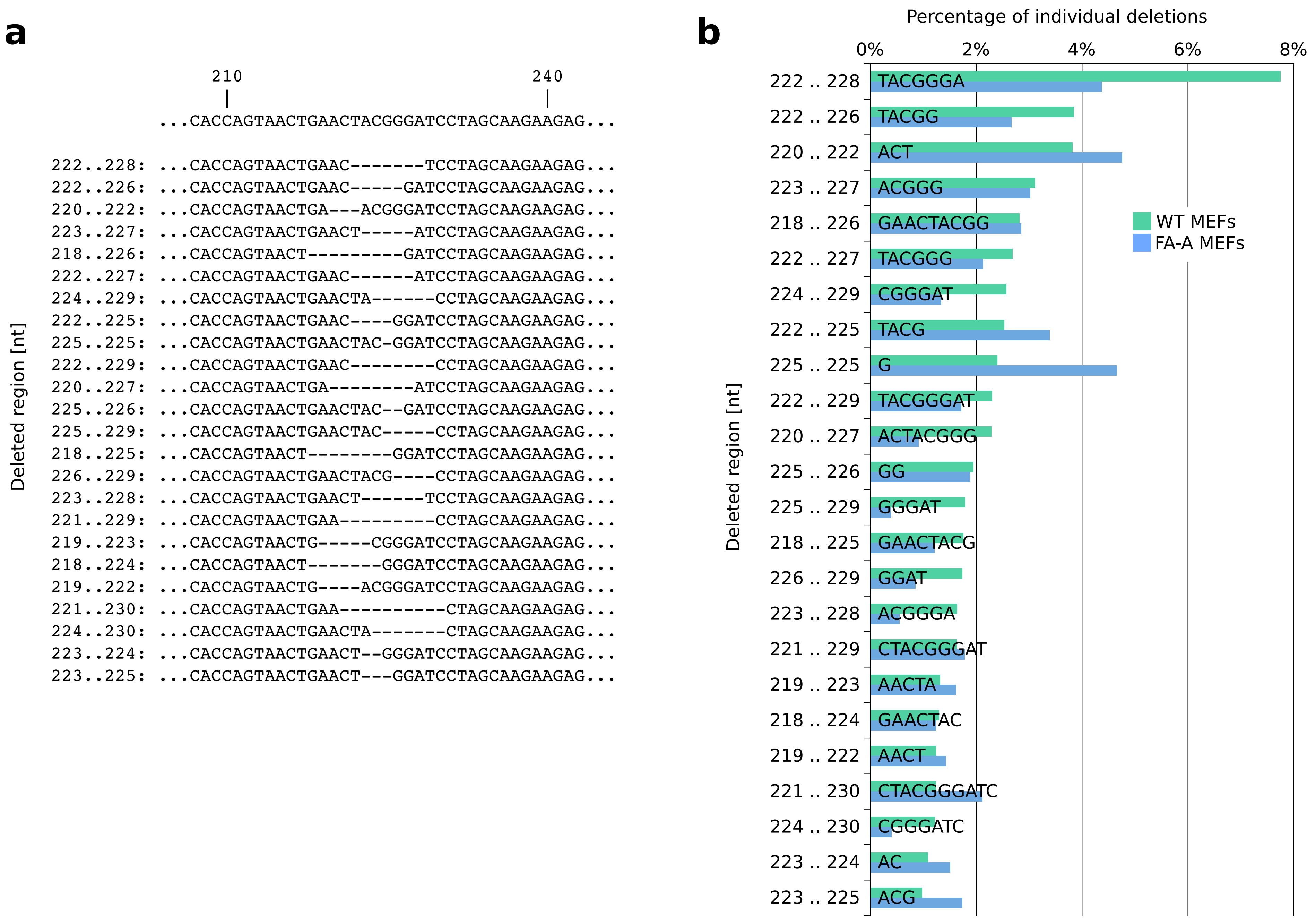

Supplementary Figure 3

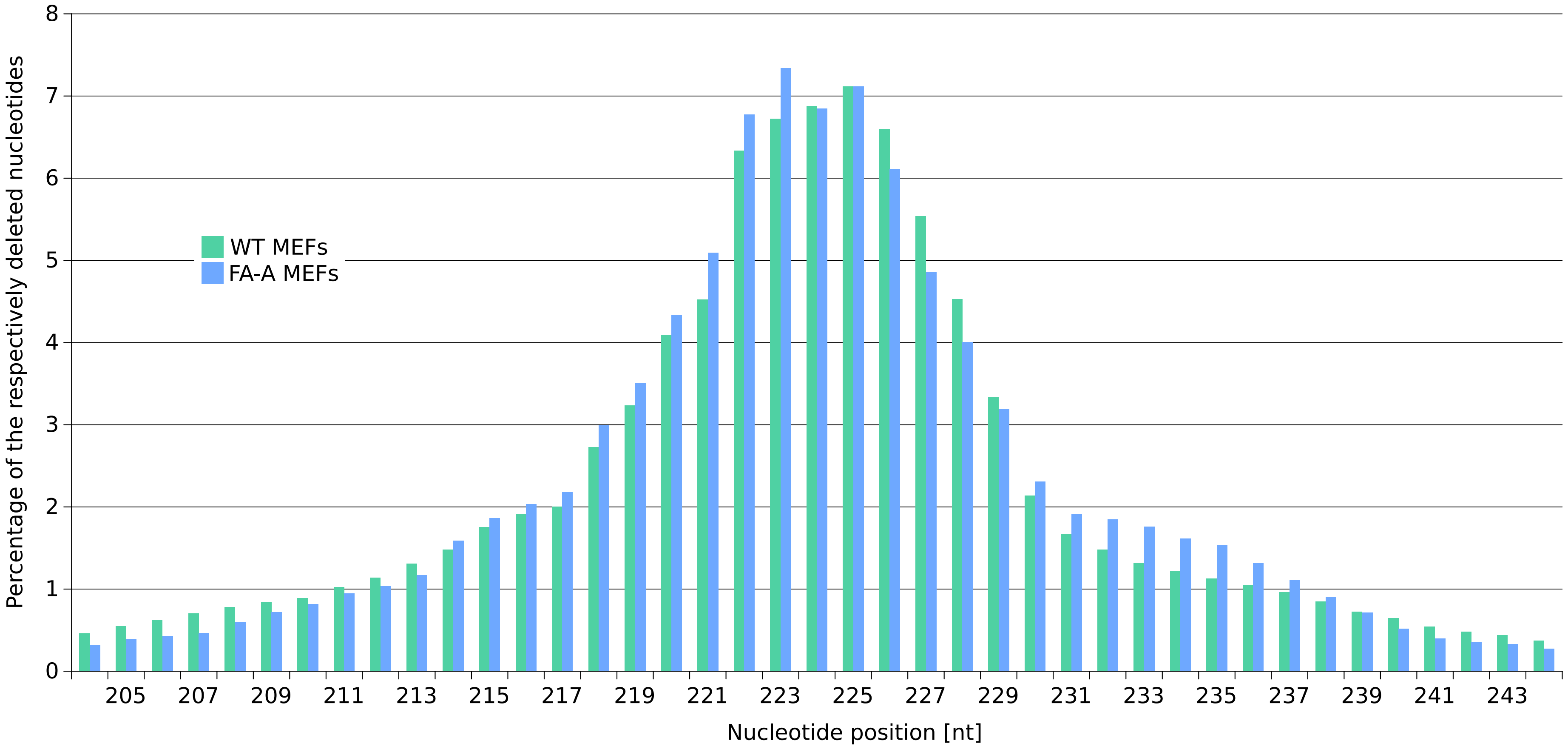

Supplementary Figure 4

A.

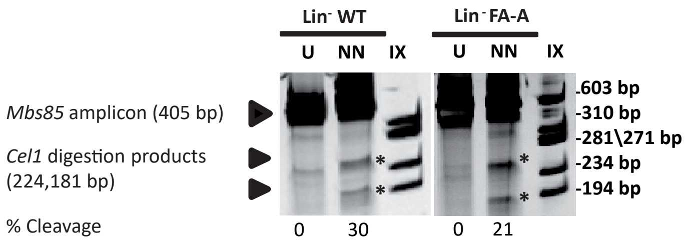

B.

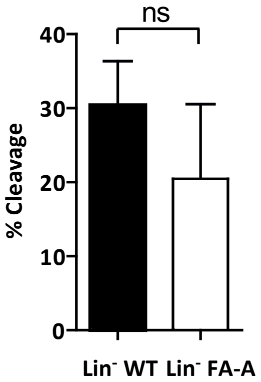

Supplementary Figure 5

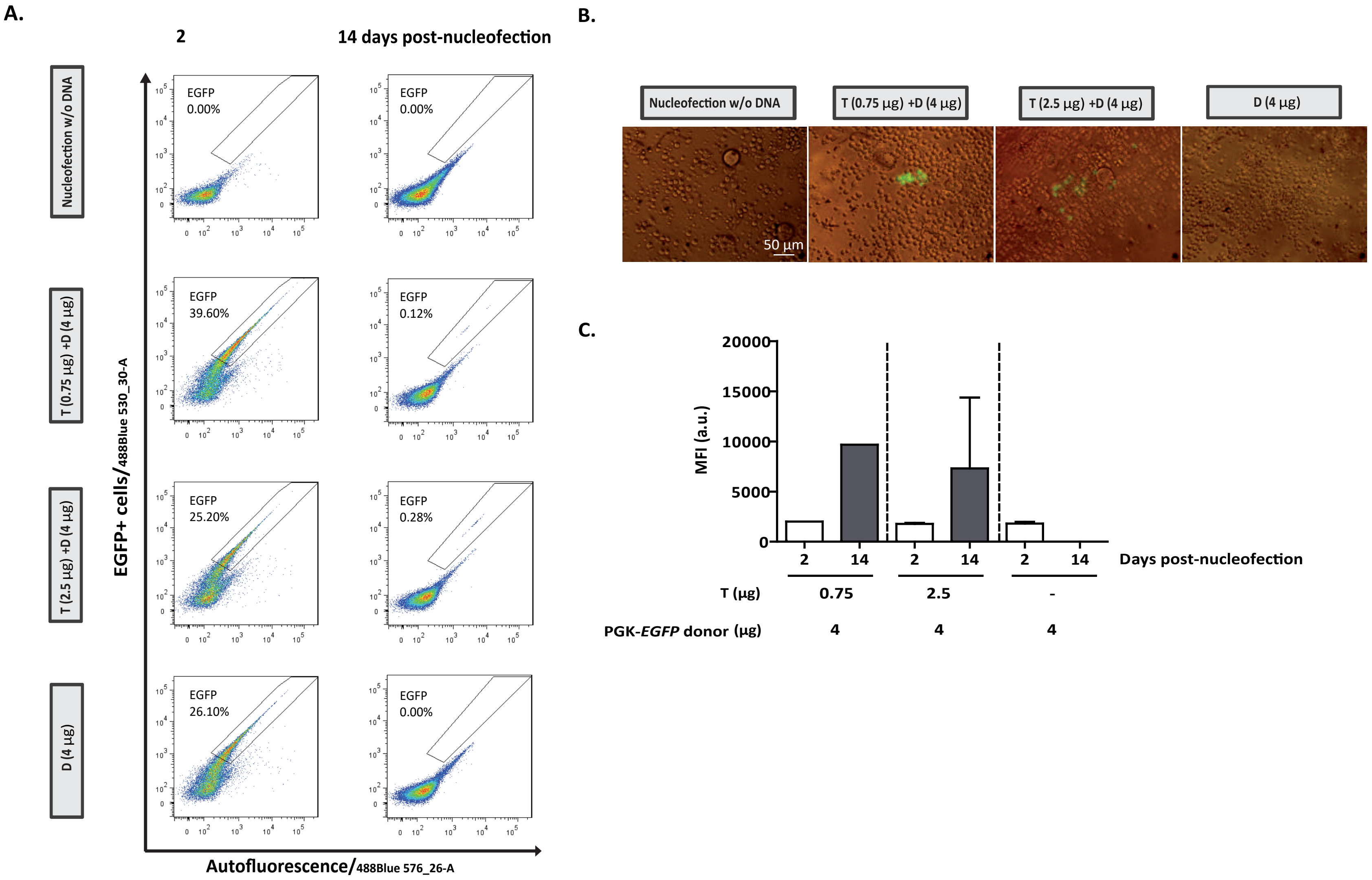

Supplementary Figure 6

Supplementary Table 1

ID	Closest Gene	Region	Chromosomal coordinates[1]	Match Type	mismatches Left	mismatches Right	Left Target (5'-->3`)	Spacer (bp)	Right Target (3'->5`)	WT MEFs indel frequency nuclease treated	WT MEFs indel frequency untreated	WT MEFs p-value[2]	FA-A MEFs indel frequency nuclease treated	FA-A MEFs indel frequency untreated	FA-A MEFs p-value[2]
1	Mbs85/Ppp1r12c	Intron	chr7:4499924-4499974	R13L	0	0	TCTGTTCCCACCAGTAACT	13	GATCGTTCTTCTCTCCTGT	37.12%	0.10%	**<0.0001**	30.15%	0.04%	**<0.0001**
2	Vstm2b	Intron	chr7:40912153-40912210	R20L	3	6	TCTGTaCCCcCCAGaAACT	20	GgTCtcTCTcCTCTCCctT	0.08%	0.07%	0.3607	0.14%	0.12%	0.0524
3	Gcnt4	Intergenic	chr13:96885014-96885067	L16R	4	4	TGaCCTCcCTcCTTGCTAa	16	aaAATGACgACaCTTGTCT	0.07%	0.11%	0.9925	0.06%	0.08%	0.8157
4	Opcml	Intron	chr9:28002385-28002449	L27L	3	5	TGTCCTCTCTTCcTcaTAG	27	GATCGgTgTTCTaaCCgGT	0.08%	0.07%	0.261	0.07%	0.08%	0.6144
5	Ush2a	Intron	chr1:188788167-188788219	L15R	4	4	TcTCCTCTCTTCTTcCcAt	15	aaAATGACgACaCTTGTCT	0.11%	0.19%	1	0.12%	0.16%	0.9931
6	Hs3st3b1	Intergenic	chr11:63609864-63609925	L24R	6	3	TGTCtTCTCTgaTTGgTta	24	TCAATGACCcCtCTTaTCT	0.07%	0.07%	0.3805	0.08%	0.07%	0.0729
7	Gm15800	Intron	chr5:121256750-121256814	R27L	6	3	gCTtTTCCCcCCAGcAgCc	27	GATCGgTCgTCaCTCCTGT	0.05%	0.06%	0.7362	0.06%	0.07%	0.8889
8	Pga5	Promoter	chr19:10678302-10678365	R26R	5	4	TCaGaTCCCACCAGctcCT	26	cCAAcGACCgtCCTTGTCT	0.06%	0.05%	0.0711	0.06%	0.05%	0.3296
9	4933402J15Rik	Intergenic	chr14:74102062-74102121	L22R	6	4	TtTaCTCTCTcCTTaCTca	22	aCAAaGACCAaCCTcGTCT	0.09%	0.09%	0.7021	0.09%	0.08%	0.154
10	Odz4	Intergenic	chr7:95688609-95688671	L25R	6	3	TGTCCcCTCTTCTTttaca	25	TCAATccCCACCtTTGTCT	0.06%	0.06%	0.532	0.06%	0.06%	0.3905
11	Acbd6	Intron	chr1:155609313-	L27L	4	6	TaaCCaCTCTTCTTGCTAa	27	GgTgGTTtcTCaCTCCaGT	0.09%	0.09%	0.251	0.09%	0.08%	0.1802

			155609377												
12	Nkx6-1	Intergenic	chr5:101585362-101585421	R22L	4	5	TCTGTTCaCAgCAGTAAaa	22	GATCGTTaaTCTtTaCTaT	0.12%	0.15%	0.9362	0.14%	0.16%	0.9359
13	Adam32	Intron	chr8:24878909-24878961	R15L	6	4	TCTaTctCCACtAtTAcCT	15	GATCaTTCaTCatTCCTGT	0.36%	0.09%	**<0.0001**	0.33%	0.11%	**<0.0001**
14	Cdh11	Intergenic	chr8:102833188-102833235	R10R	6	3	TCTGTaaCCtagAaTAACT	10	TCgtTGACtACCCTTGTCT	0.06%	0.07%	0.9009	0.08%	0.06%	**0.0195**
15	Gm5072	Intergenic	chrX:91403414-91403472	L21R	5	4	TGTaCTCTtTgCTTGgTgG	21	TCAATGACCAaCCaaGTaT	18.37%	19.34%	1	17.00%	18.10%	1
16	Cry1	Intergenic	chr10:85202507-85202573	R29L	4	5	TCTGTaCaCACCAcTcACT	29	GtTCGaTtTTCTCTCCcGa	0.14%	0.14%	0.5045	0.13%	0.13%	0.4995
17	Il20ra	Intron	chr10:19752347-19752396	L12L	4	5	TtTCCTCTtTTCTTtCaAG	12	GATCcTTtTcCTCTCCTca	0.11%	0.08%	0.0559	0.07%	0.12%	0.998
18	Sec11c	Intergenic	chr18:65798262-65798317	R18R	5	4	TCTGTcCaCAaCAGTAtaT	18	aaAATGACgACaCTTGTCT	0.06%	0.07%	0.7833	0.08%	0.07%	0.4279
19	Tmem132b	Intergenic	chr5:126222277-126222336	L22R	6	4	TaTCCaCTCaTCTTtCaAt	22	TCAATGAtgACaCTTaTCT	PCR failure					
20	Atp6v1h	Intergenic	chr1:5298497-5298562	L28R	4	4	TGTagTtTCTTCTTGCTgG	28	TCAActACCACCCTTGgtT	PCR failure					
21	Rab2a	Intergenic	chr4:8505300-8505349	R12R	6	4	TtTtTTCCCAaaAaTAACa	12	aCcATGACCACCCgTGTaT	0.24%	0.18%	**0.0034**	0.19%	0.17%	0.1491
22	Luc7l	Exon	chr17:26254092-26254159	R30L	3	6	TtTtTTCCCACaAGTAACT	30	agTCaTTtTTCTgTgCTGT	0.10%	0.23%	1	0.12%	0.12%	0.5934
23	Akr1b7	Intron	chr6:34420642-34420698	L19L	4	6	TGTCtTCcCTTCTTcCaAG	19	GATCtTTCgaCcCTaCTGa	0.05%	0.05%	0.4947	0.04%	0.05%	0.5552
24	Pvt1	Intergenic	chr15:62406674-62406723	L12R	6	4	TGTCCTtaCTTCagGaTgG	12	aaAATGACaACaCTTGTCT	0.12%	0.11%	0.3355	0.10%	0.11%	0.7653

[1] Chromosomal coordinates are based on the mm10 genome, [2] In bold the sites with a statistically significant differences as compared to controls ($p<0.05$).

Supplementary Table 2

ID	Closest Gene	TALEN Score	Forward Primer	Reverse Primer	Amplicon length	Predicted cleavage site	Amplicon Sequence
1	Mbs85/ Ppp1r12c	100	GGACGGATGGATTCTGGGTG	GGGTTCTTCTGGATTCAGGATGC	405	225	TCACCTTGCTTCCACTTTCCAAGCCCCTGGTTTCCTGAGCTCTAAGTACCCACTCTACCACCCAAGCTACATCGCAGCCCAGGACGGATGGATTCTGGGTGTTTTTTCTCTACCTGCAAGTGTGAAAATTGAACCTGTCAAATACCCTCGCTCTGTTTCCCATCTCCACAGGCAGCAGGCGTCCCTTCCAGGATCCCCTCTGTTCCCACCAGTAACTGAACTACGGGATCCTAGCAAGAAGAGAGGACAACCCAGGAGATGGAAGTTGCCATGAAAGAAGCTGCCCACCAGAAGACTGACATTGGACCAACACTGAGACCAGGAGGGAGAACTGGGATGTAGGCTCTTGTGTGACTTATCTTGTTCTCGGTCATCCTAACTGAATAAAAGCATCCTGAATCCAGA
2	Vstm2b	56.69	GGCCAGAGGGAAGTTCAACTAC	GGATGGCGTGAAGATGAGGAC	369	234	GGCCAGAGGGAAGTTCAACTACTTTTCTACACAAGAATTCTCCTTTCAAATACACCAGTAATGCCTGTTGTGTTTGAAGTTACCACTAGACTTTCTTTGGAAATTCAAAGATGATTTGGACAGGATACCTGACCAAGCACTGAGAAGTTTAAGATAACCACAAAAGTATAAAGGAGAGGGAGAACACTCGAGTTAGAGGACAGTCTGTACCCCCCAGAAACTCATTGTAGAGACACTAGAGCCCAGAGAGAGGAGAGGGAAAGGACAAGGGAAGGAGAGCATCAGACCCTCAGAGAGAGGAGAGGGAAAGGACAAGGGGAAGGAGAGCATCAGACCCTGATCCTGCTAGTCCTCATCTTCACGCCATCC
3	Gcnt4	55.89	GTCTCTTGGACTACCCTGACTAAC	CTGAATAGATACTGCCATGCTCCC	292	156	GTCTCTTGGACTACCCTGACTAACAACTCAAAAAGAGGTTTCATTCAAGGTGTGTGTGTGTGTGTGTGTGTGTGTGTGTGTGTGTGTGTGTGTGTGTGTTACTCATTCCCACTCCTTTTCTTTCTGTATTGACCTCCCTCCTTGCTAAGCTGTCTTAGTTAGGGTTTTACTGCTGTGAACAGACACCATGACCAAGGCAAGTCTTATAAAAACAACATTTAATTGGGGCTGGCTTACAGGTTCAGAGGTTAAGTCCATTATCATCAAGGTGGGAGCATGGCAGTATCTATTCAG
4	Opcml	55.47	GTTTTTGTTCCCTGACACCCTGAG	GACTCTGCCAGGTGCACTGT	283	147	GTTTTTGTTCCCTGACACCCTGAGTTATTATCATAGTGGTGGCAGATACTGTTACCTAGGTCTTAGCAAGGCAAAGGCTGATAGGTGAGAAAGAGACTTACGTGGTTTCCTGGTGTCCTCTCTTCCTCATAGGAAAGTGGATCTCTTTCTTTGTGTTAGCTAGCCACAAGATTGGCCATAGCCTCAACCTAAAGCCAGCCTCGTCTGCTCTCATCACATCCAGAGAATATGGCCAGACACAGAGCCCCACAAAACCCTTTTGGACAGTGCACCTGGCAGAGTC
5	Ush2a	55.04	CAGGGGACAACTTTTGAGTGTCCA	CTCAGCTCCTCCTGCACCAT	295	150	CAGGGGACAACTTTTGAGTGTCCATTCTTAGTTCTCTGCAGATTGATGCAGGGTCTCTCCCTTTTCTGTCACCCCATAGCATACTCCAGGATAGGTGGCCCATCAGCTTCTGGCCAAGTCTCTCTCCTCTCTTCTTCCCATCTGTCTTAGGTAGGGTTTTACTGCTGTGAACAGACACCATGACCAAGGCAAGTCATTTAATTGGGGCTGGCTTACAGGTTCAGAGATTCAGTCCATTATCTTCAAGGTGGGAGCATGGCAGTATCCAGGCAGGCATGGTGCAGGAGGAGCTGAG
6	Hs3st3b1	54.7	GGAGCATTTTTGGTGCTGCTGTG	CCCATCACATCTTAGAGAGACCC	278	143	GGAGCATTTTTGGTGCTGCTGTGTATTCAAAGGCTAAGTACCAGCCCCCCATAATCTGTGTGCCCCTTGAACAGATCCTCAGGTACACCGAGTGAGGGGTAAACGGTCCTTGTCTTCTCTGATTGGTTAAATACACAGATGTGGCTGACAGCCAGTTACTGGGGAGAATAGAGGTAGGTGAGGCTAGGTTCCTGGGCTCAGCGTCCTAGGTAGGGACCACAAGGAGAGAAGGAGGAGGAAGAAGAAAGAGGAAGGGGGTCTCTCTAAGATGTGATGGG
7	Gm15800	54.44	CCCTCTCCAGCAGTTATGCC	GGAACAAAGCTGAGATGGGACAAG	274	136	CCCTCTCCAGCAGTTATGCCATTATTGCACCAGTGAGGACCATCCTGCCTGGCAGGTGAGTAGGGTGTGCTTAGGCTTCACAGCTGGATGGGACTGTTGATGGCTTTTCCCCCCAGCAGCCCACCTGGTACTGCCAGCACTGTGAAAGCTAGCCAGCAGTGAGGACACTTCCAGTTTCTCCTGCATTTGGGGGAGAGAGTGTGTGTGTGTCTCCCTATGTAAGCCAGGGACGTCAGGGAAGGAGACCATGCTTGTCCCATCTCAGCTTTGTTCC
8	Pga5	54.24	GGTAGCACCAAGTCACCTGATTTC	GCAAGGACTAGCTGCCTATC	**381**	144	GGTAGCACCAAGTCACCTGATTTCACAGAGTGGAGAGACTCTTGCTATCTTGGTGATAAAGACACTGTGGATAAAGTGACAAAGGTAAGTGCCAGGTGCCTCTCTGCTGGTCAGATCCCACCAGCTCCTCACTGGCCTGTCCTCATTAGAGA

				TTC			GTGGGTTGCTGGCAGGAACAGAAGGCCGAGTCTGACAACCTGAGTTTGATAGCCAGGGCCAAGTTTAACCTCCATACACCTCTGTGGTAGAGGCACCCACCCACCCACATCAAACAAACAAACACATAAATACAACAGTAAATGTAAAATGTATCCATGATAAGTGGAAGACTTTTAAAAAGTTTATTTTTCTTTAAAAACCAAAAGAAGATAGGCAGCTAGTCCTTGC
9	4933402J15Rik	54.16	CTCATGAGTTTCTGGGAGAGGTAG	CCACTGTGGATGATGCTTCCTAAC	**395**	224	CTCATGAGTTTCTGGGAGAGGTAGAAAAGTAGTCCCTAAAAGTTAAGCTTTCAAAAGCAACAGTGAAAGCTATGTTGCAAAGTAACCTAAAAAGGTGTCAGCTGTTGCTGTAGCTACCTGTCTGTGATTAGTGCTTCTATAGCTAGAGTCTTCTACTGACATCTCTGCTACCAACGCAGATGCTCAAAAAGCTTTACTCTCTCCTTACTCAAACAGTGGAAATCCCATGTAGGTGTTTCTGGTTGGAGCAGAAATAGCATGGAGACCTAGAAAATCATAACAGTGTAAGAAGGATGTTCATTGCGTTAGTCATATAAGCCAGGTGTTCACACATGTCTTTGTAAAACTATCGAGATTGTCAAATGAGTTGTGTTAGGAAGCATCATCCACAGTGG
10	Odz4	53.96	CAGAGTGCTCCTCAACCTGC	CACACAAAGCTCAGCCTGAAGAC	308	137	CAGAGTGCTCCTCAACCTGCTCCACTGCCCTGCATTCACAGCTAGCCATCACCCCTCCACCCCCCAACACCAAGTGAATGAATCGAAACCTTGGGTAAGGGGAATGTCCCCTCTTCTTTTACATTTGAACTCAGCATCAATTTCATCCAGTTAGGGGTGGAAACAGAAAACCGAAAAGAAAAGAGAGAGAGGGACTCACCAGGATGTTGAATGGGCAAAAGAAGAAAGAATAGAAAGAAAGAAGGAAAGAAAAAGAAATCTCTCAAAAGGCACTTTTATTTCATTGTCTTCAGGCTGAGCTTTGTGTG
11	Acbd6	53.45	CTGCCGTGCAGTAGCTTTCTAG	CCATGTTCCCCACCGTAACTG	**341**	140	CTGCCGTGCAGTAGCTTTCTAGAATATGTTCCTCCAGTGTAACCAAACTTTGTTGCTTTAACCAACATCTTTGTTTCTGACCTGTCCTCCCTCTTCTAGTCTGAGGTAACCACTCTTCTTGCTAAATTGATGTGAAGTGTACCTTTAAGAATCCACCAAAGAGTGAGGTCATACAGTGTCCTTCTGTGTTAGACTTCTTTCCCGCGTCATAGTGTCTTACATACGGCTCTATTGCTGTGAAGAGACACCATGACCAAGGCAACTTATAGAAGAAAACATTTAAATGAGGTTTACAGTTTCAGATGATGAGTCTACTATTACAGTTACGGTGGGGAACATGG
12	Nkx6-1	53.39	CCGCTGCCATAACTCAGCCT	GGGATGGGAGGTGAATATGTCCAA	**353**	140	CCGCTGCCATAACTCAGCCTTGATGATAATAATGAACTGAACCTCTGAACCTGTAAGCCAGCCCCAATTAAATGTGGCCCTTATAAAAGACTTGCCTTAATCATGGTGTCTGTTCACAGCAGTAAAACCCTAACTAAGACAGGTTGTTTCTAGCAATTAGAAATGATATTATTTCTATGGTAGCCATCAGTAATGAGCAAACATCTTAATTATTGTTCTGCTGTGTCAACTGATTCTGAAAACGATCATTTTGTCTTAGTATTCATTGTGTGTGTAATATTGTTATTCTACATGTATATAGAATAACATAATACATATGATAATTTATTTTGGACATATTCACCTCCCATCCC
13	Adam32	53.38	CCCTGGATTTCTTCATAGCAGGC	GCCTTGTGCTCTACATACACATCC	289	150	CCCTGGATTTCTTCATAGCAGGCAAATGGAGCATTCACTGAACCTATCAAAATTAGAAACATATTTAGATTAGATTATATATTTGTGCTTCTCCATGTCCTCATACACCATGCTTCCTTACCTCTATCTCCACTATTACCTAACTCAAAAGGTTTTCTAGTAAGTAGTAAGGACAGAGAAAGAACTTTCATGACTATGATGTAATGCTACTTTGCTACATTGTATTCATTTCTGTAACATAAAATACAAGAGAAAGGAGAGTTATGGATGTGTATGTAGAGCACAAGGC
14	Cdh11	52.96	GGCAGGGCTAAAGACAGAGCA	CTCTGTGAAGCCTGGTGAGTTCAA	**320**	152	GGCAGGGCTAAAGACAGAGCAAAATTTGAGAACTTGGTCAACCAATGACTGACTCAGCTTGAGCTCCATGCCATGAGATGGAGATCACCCCTGACACTGTTAATGACACCCCTGCTATATTTGCAGTCTGTAACCTAGAATAACTGGGTTCATCCAGCAACTGATGGGAACAGATACAGAGAACTACAGCCAACCATTAGGCAGAGCTTGGAGCATCCTTCAGAAGAAGGGGAAGAAGGATTGTAGGGGCTAGAGGAGTCAAGGACAACACAAGTATACTTACAGAATTGACTAATTTGAACTCACCAGGCTTCACAGAG
15	Gm5072	52.87	GTGGAGCAGAGAGCCTTCACA	GGATGCTCTCAGCCAACCATTGAA	271	135	GTGGAGCAGAGAGCCTTCACAGGACCAAGGACCACTCCTCCCATTGATGTCCAAGAAGGCCATCCTCTACTACATATGCGGCTAGAGACATGGGTCCCTCCATGTGTACTCTTTGCTTGGTGGTTTAGTCCCTGGGAGCTCTGGAGTTACTGGTTGGTTCATAATGTTGTTCCTCCTATAGGGCTGCAAAACCCTTCAGCTCCTTAGGTCCTTTCTCTAACTCCTCCATTGGGGACCCTGTGATCAGTTCAATGGTTGGCTGAGAGCATCC
16	Cry1	52.82	CCCCCCCTTTTTAGAATGTACTGC	TCCCAGTCTTCCTTCTTGAAC	301	165	CCCCCCCTTTTTAGAATGTACTGCTAGATTTATCAACAGAATAAGAAGATCAATCTCTTGAGGCTTACAAGAGAGACTGCAGTTTACATTTCCCCACTGCAATGCATCGAACGGAAGTTTAAAGGTGATCTCTGTACACACCACTCACTTCCCAG

				CC			CCCCTTTTCCTTCTCACAGACAGCAAGCTAAAAGAGAGGGCTGTAAGTTCTTTCCTCCCTAAGAGTGTTCATTTTCAGCAGAATTGGCAAAGAGGGGGGAACCCTGCTTGCAATGCAATCTGAGGGTTCAAGAAGGAAGACTGGGA
17	Il20ra	52.81	AACCCACAGTCCCAGCTCCA	CCTTCCCTGCTTGCTTGGTATTTG	285	136	AACCCACAGTCCCAGCTCCACCCCCACCCGTCTCAACTGCACCCCTGCCACAACCCCACTCCCACCCCAGCCCCAGCCTCACCCCTATCCCCACCCCTTCCCACTCTACTTTCCTCTTTTCTTTCAAGTTGAAACAGTCCCTAGGAAAAGGAGAGGAGTGTTGGAAACACATTTGAACAAGGAAAAGCTTTCCTTCTGAGTTACTTTATCATGCCGTCTCTTTGAAAAGAAAGCATTCTCTACCATAATGCAAATAAAACTCAAATACCAAGCAAGCAGGGAAGG
18	Sec11c	52.77	AGGCACACCGTGAGCCTACA	CTACCTGGATACTGCCATGCTC	272	137	AGGCACACCGTGAGCCTACAGGTGGTGGGGCTGGCAGAAGCATCAGGGACAGGAAGGCAAATCCATAAGCAGATCATATGACGATATCTGGGAGCATAAATCTTTGCTCTGTCCACAACAGTATATCCAGTGTCTTAGTTAGGGTTTTACTGCTGTGAACAGATATCATGACCAAGGCAAGTCTTATAAAAAACATTTAATTGGGGCTGGCTTACAGGTTCAGAGGTTCAGTCCATTATCATCAAGGTGGGAGCATGGCAGTATCCAGGTAG
19	Tmem132b	52.72	PCR failure	19			
20	Atp6v1h	52.56	PCR failure	20			
21	Rab2a	52.53	CTGACAGTGCTGTCCTGTGTATAC	CTGCTGGCTATCTTTGACAGTGC	363	222	CTGACAGTGCTGTCCTGTGTATACAAGCATAAATATTTAAAAGGCAATTTGGTGGCGTATTATGTCCAAATAACAGCAGAAGAATCCCAATTAAGGTCTATAACATTCAACCACAGGCTTTTCACTGGGTTTCTAGTACCAGATGTGAATTCATGCCTGTGGAGTGGGCCTCAAATTCAATACAAAAGCAGTTGTTTTTTTCCCAAAAATAACAGTCATGCAACTATGGTACTGGTGGGCACATATTGCCTGGAAGGTGGGTAGTCTGGCATGCAGAAGCCACAGCTGAGTCAGACTGTTGATGACAATTCTCCCCCAGAAGCCTACACAGACCCTTCTAGCACTGTCAAAGATAGCCAGCAG
22	Luc7l	52.44	TGCCTACATCAATCTGCAAGGGAG	GCAGTAAGGAAGTCGGGGAATG	311	136	TGCCTACATCAATCTGCAAGGGAGTTGCAGAAAAGCCTCATGTTCATCGAGCCGTGAGTCACAACCAATTTCTAAGCTGTTATAACAAAAAAGTGTTTGCTTTTTTCCCACAAGTAACTTTAAAAGTGTAGTTTAGAAAGAAAACATTTTCAGTAAAAAGACACGACATTAATCCTGGATGCTTGCCAATCCTGAAGTATATTCTCCCTGACTATTACACAGCACTGTGTCCTGTACACAGATAGCCTTAGAATTTGTCACATACCACTTTGCCTTTACTTTTATGTATCATTCCCCGACTTCCTTACTGC
23	Akr1b7	52.43	CTGGGCTGTACATCTGACATTCC	GCTAGCACAGCATTCCACCTATGA	281	135	CTGGGCTGTACATCTGACATTCCTCAGTGGAACAGGTGATAGGAGGGAAGGAGGACTCTTCTTCCCTGCCCTTCAGCTTCTCATCCCACTCTTTCACTGACAGTGTGTCTTCCCTTCTTCCAAGCACTCTGTCAACTTGTAGGCTAGAAAGCTGGGATGACTGAGCTCACAGGTGGTACCGGCCAAACCTGTCAGGACTGATCACAACTGTAGCTTATATCACTGAGATGACCACTGCCTACCATTGAGCACTTAGATCATAGGTGGAATGCTGTGCTAGC
24	Pvt1	52.4	GAAGCCAAAGGAAGGGCAGG	CTTCCTGGATGCTGCCTTGTTC	310	175	GAAGCCAAAGGAAGGGCAGGAATATTTTTGTACCCTTGAATGCAATTAAATTCTAAAGCAGGTGTGAGGCTGAACTCTAGAACACAGCTTCCAACCCAGAGAAGGATACCTTGGACCTTCCTCTTTTTCCTCAGTCTGTAGAAGATACTGTCCTTACTTCAGGATGGTCTTAGTTAGGGTTTTACTGTTGTGAACAGATACCATGACCAATGTAAGTCTTATAAAGTACAACATTTAATTGGGGCTGACTTACAGTTTCCAAGATTCAGTCCATTATTGTCAAGGCAGGAACAAGGCAGCATCCAGGAAG

Supplementary Table 3

		WT MEFs						FA-A MEFs					
		Untreated			Nuclease treated			Untreated			Nuclease treated		
ID	Closest Gene	Total number of aligned reads	Reads with indels	Indel Frequency	Total number of aligned reads	Reads with indels	Indel Frequency	Total number of aligned reads	Reads with indels	Indel Frequency	Total number of aligned reads	Reads with indels	Indel Frequency
1	Mbs85/Ppp1r12c	103938	109	0.10%	128806	47810	37.12%	107317	43	0.04%	97966	29534	30.15%
2	Vstm2b	120329	87	0.07%	110015	84	0.08%	81881	96	0.12%	105707	153	0.14%
3	Gcnt4	69690	74	0.11%	65566	44	0.07%	45999	37	0.08%	40663	26	0.06%
4	Opcml	91141	64	0.07%	55282	44	0.08%	117583	90	0.08%	95791	70	0.07%
5	Ush2a	100840	189	0.19%	78141	84	0.11%	90515	142	0.16%	113998	133	0.12%
6	Hs3st3b1	197005	134	0.07%	291378	205	0.07%	176089	120	0.07%	425033	338	0.08%
7	Gm15800	95634	54	0.06%	107987	54	0.05%	102713	69	0.07%	150677	83	0.06%
8	Pga5	115423	57	0.05%	91053	59	0.06%	38234	21	0.05%	43245	27	0.06%
9	4933402J15Rik	74900	70	0.09%	72764	62	0.09%	84815	67	0.08%	75574	71	0.09%
10	Odz4	195818	120	0.06%	201147	122	0.06%	200030	124	0.06%	185237	119	0.06%
11	Acbd6	92628	79	0.09%	83253	79	0.09%	86611	70	0.08%	76424	72	0.09%
12	Nkx6-1	95298	141	0.15%	87883	107	0.12%	102318	166	0.16%	88023	119	0.14%
13	Adam32	104306	93	0.09%	108741	396	0.36%	97994	108	0.11%	123054	403	0.33%
14	Cdh11	93757	69	0.07%	94248	55	0.06%	90752	51	0.06%	85379	70	0.08%
15	Gm5072	156935	30349	19.34%	145673	26758	18.37%	140998	25520	18.10%	142535	24233	17.00%
16	Cry1	66067	94	0.14%	71101	101	0.14%	67634	89	0.13%	72181	95	0.13%
17	Il20ra	71316	57	0.08%	77064	81	0.11%	76799	93	0.12%	63527	46	0.07%
18	Sec11c	73213	48	0.07%	84174	47	0.06%	75950	56	0.07%	61495	47	0.08%
19	Tmem132b	PCR failure											
20	Atp6v1h	PCR failure											
21	Rab2a	63093	112	0.18%	81971	200	0.24%	69384	118	0.17%	64857	126	0.19%
22	Luc7l	178913	413	0.23%	196906	199	0.10%	235926	283	0.12%	188185	221	0.12%
23	Akr1b7	111556	56	0.05%	95369	48	0.05%	121969	55	0.05%	104876	46	0.04%
24	Pvt1	112860	124	0.11%	113056	131	0.12%	116172	125	0.11%	111365	109	0.10%

Supplementary Table 4

Purpose	Name	Sequence (from 5' to 3')	Tm (ºC)	Product size (pb)	Fluorochrome
qPCR *Sry*	Sry-F	TGTTCAGCCCTACAGCCACA	53.9	140	------------------
	Sry-R	CCTCTCACCACGGGACCAC	54.8		------------------
	Sry Probe	ACAATTGTCTAGAGAGAGCATGGAGGGCCA	64.7	------------------	6-FAM
qPCR *β-Actin*	β-Actin-F	ACGGCCAGGTCATCACTATTG	53.9	131	------------------
	β-Actin-R	ACTATGGCCTCAGGAGTTTTGTCA	55.9		------------------
	β-Actin Probe	AACGAGCGGTTCCGATGCCCT	63.5	------------------	Joe
PCR *T7E1* or *Cel1* assay	mAAVS1 CelIF	TCTGGATTCAGGATGCTTTT	54	405	------------------
	mAAVS1 CelIR	TCACCTTGCTTCCACTTTCC	58		------------------
5' PCR Integration junction	mAAVS1-5’F1	TTGTGGCCTCAGGACAGTGTAC	64	1167	------------------
	mAAVS1-5’R1	AACGGACGTGAAGAATGTGCG	61		------------------
	mAAVS1-5’F2	GGTTTCGTTCTCCTGCACTC	60	1439	------------------
	mAAVS1-5’R2	GTCCGTCTGCGAGGGTACTA	63		------------------
	mAAVS1-5’F3	TTCCCGTGACTTGTGCTGTA	64.4	1029	------------------
	mAAVS1-5'R3	CCACGGGGTTGGGATTATTAT	65.2		------------------
3' PCR Integration junction	mAAVS1-3’F1	ACAGATGGAAGGCCTCCTGG	63	1395	------------------
	mAAVS1-3’R1	TCTTGGAACTTTCACTGCTAAAGC	62		------------------
	mAAVS1-3’F2	GCAACCTCCCCTTCTACGAG	63	1337	------------------
	mAAVS1-3’R2	GATGCCCCAAGGAGGGTTTA	60		------------------
	mAAVS1-EGFP-3'F	GTGGTTTGTCCAAACTCATCAA	63.7	1035	------------------
	mAAVS1-EGFP-3'R	TCCTTGTTTTCCTGGGACT	61.1		------------------
PCR for sequencing 3' integration junction	mAAVS1-3’F2	GCAACCTCCCCTTCTACGAG	63	------------------	------------------
	mAAVS1-3’R2	GATGCCCCAAGGAGGGTTTA	60	------------------	------------------
	SequencingAAVS1-1_3'_F	TGGAGATGGGAAACAGAG	58.7	------------------	------------------
	SequencingAAVS1-2_3'_F	TGTCCCTAGAAGCTCTGGTG	62	------------------	------------------
	SequencingAAVS1-3_3'_F	CTGACTGCATCCCTCTCCTC	64	------------------	------------------
	SequencingAAVS1-4_3'_F	GCTTGGAAAGTGGAAGCAAG	63.8	------------------	------------------
	SequencingAAVS1-1_3'_R	TTGCATCTCCTTTCCCAATC	63.9	------------------	------------------

Supplementary Figure 1: Efficient TALEN-mediated editing of *Mbs85* locus. A) Schematic representation of the *Mbs85* genomic locus (Chromosome 7: 4,481,520-4,501,680) and the TALEN-targeted sequences (intron 1). The TALEN backbone contains an N-terminal NLS, the '0 repeat' , the 17.5 'half-repeat'. This is followed bythe C-terminal domain fused to the catalytic *FokI* cleavage domain, as shown in panel B) The amino acids sequence of the DNA binding modules of the engineered TALENs (represented with different colors according to the cipher NG = T, HD = C, NI = A and NN = G or A), as well as the expected target sequences are indicated. **B)** Evaluation of TALEN expression in HEK-293T cells by WB analysis using an antibody directed against the HA-tag present in each TALEN monomer. **C)** Evaluation of the cleavage efficacy of the *Mbs85*-specific TALEN pair using the mismatch sensitive Surveyor assay in WT and FA-A MEFs. Representative electrophoresis gel showing the frequency of indels (calculated as the mean percentage of modified alleles) at the target locus using 2.5 µg of each TALEN monomer. Arrows indicate the size of the parental band (405 bp) and the expected positions of the digestion products (224 bp and 181 bp), that are also indicated with asterisks. ϕ: transfected cells without DNA (mock condition); IX: DNA molecular weight marker.

Supplementary Figure 2: Differential repair preference leading to insertions in WT and FA-A MEFs upon nuclease treatment. Positions of insertions and respective sequences identified at the on-target site. The 24 top most insertions in the on-target locus are shown in **A**) as alignment to the reference sequence, and highlighted in bold and in **B**) as percentage of all insertions comparing WT-MEFs to their respective counterparts FA-A MEFs. Nucleotide positions refer to the amplicon sequence provided in Supplementary Table1.

Supplementary Figure 3: Differential repair preference leading to deletions in WT and FA-A MEFs upon nuclease treatment. For all single deletions, the deleted regions and sequences identified at the on-target site. The 24 top most deletions in the on-target locus are shown in a) as alignment to the reference sequence and in b) as percentage of all deletions comparing WT-MEFs to their respective counterparts FA-A MEFs. Percentages of individual deletions were calculated in respect to all deletions. Nucleotide positions refer to the amplicon sequence provided in Supplementary Table1.

Supplementary Figure 4. On-target cleavage predominantly occurs in the range of nucleotide 222 to 226. The deletion frequencies of each single nucleotide were calculated as percent of the total reads showing an indel in the analysed on-target region. Nucleotide positions refer to the amplicon sequence provided in Supplementary Table1.

Supplementary Figure 5. Evaluation of cleavage efficacy of TALEN in the *Mbs85* locus of WT and FA-A Lin$^-$ BM cells by Surveyor assay. A) Representative electrophoresis gel showing the disruption of the target locus using 2.5 µg of each TALEN monomer indicated as T. The extent of cleavage measured as the mean percentage of modified alleles is indicated below the image. Arrows indicate the size of the parental band (405 bp) and the expected positions of the digestion products (224 bp and 181 bp), that are also indicated with asterisks. U: untransfected cells; IX: DNA molecular weight marker. **B)** Histogram representing the percentage of cleavage monitored with the Surveyor Assay in WT and FA-A hematopoietic progenitors. Bars indicate the mean ± SD (n=3 experiments in WT cells and n=4 experiments in FA-A cells). No statistical difference between groups was observed using a Student's t-test.

Supplementary Figure 6. Evaluation of the efficiency of EGFP expression of WT Lin$^-$ BM cells nucleofected with the TALEN and the PGK-*EGFP* reporter donor at 2 and 14 days post-nucleofection. A) Representative flow cytometry dot plots of EGFP$^+$ cells analysed at day 2 and 14 post-nucleofection of WT Lin$^-$ BM cells. T+D: 0.75 or 2.5 µg of each TALEN monomer together with 4 µg of the PGK-*EGFP* reporter donor; D: 4 µg of the PGK-*EGFP* reporter donor. EGFP$^+$ expression was determined discarding autofluorescent cells (576/26 channel). **B)** Representative image in liquid culture of EGFP$^+$ Lin$^-$ BM cells at 14 days post-nucleofection. EGFP$^+$ fluorescent cells could be observed in the microscope in T+D conditions. Scale bar represents 50 µm for all the microphotographs. **C)** Geometric Mean Fluorescent

Intensity (measured in arbitrary units, a.u.) of nucleofected cells analysed in the same conditions shown in A). Data show the mean ± S.D. (n=2-3 experiments). Statistical analysis could not be performed.

Supplementary Table 1. Off-target cleavage of Mbs85-specific TALENs. The top off-targets are listed according to the ranking generated with TALENv.2 algorithm from PROGNOS software. The nucleotide differences in the left and in the right targets, the closest gene, the region and chromosomal coordinates of the off-target location are indicated. The match type and the sequence of the left and the right target are also included. The indel frequency and p-value are also indicated.

Supplementary Table 2. Details of the off-target analyses. The top off-targets are listed according to the ranking generated with TALENv.2 algorithm. The closest gene, the TALEN score, the forward and reverse primers sequence, and the amplicon size and sequence are indicated, as well at the predicted cleavage site.

Supplementary Table 3. Results of the next generation sequencing. The top off-targets are listed according to the ranking generated with TALENv.2 algorithm. The frequency of indels generated in each locus could be observed in WT MEFs and their respective counterparts, FA-A MEFs.

Supplementary Table 4. Primers and probes used in this study. Forward and reverse primers as well as probes, their sequence, their melting temperature (Tm) and the product size is indicated. In the case of probes, the fluorochrome to which each probe was conjugated is also indicated.
